# Sexual Differentiation Is Coordinately Regulated by *Cryptococcus neoformans CRK1* and *GAT1*

**DOI:** 10.3390/genes11060669

**Published:** 2020-06-19

**Authors:** Kuang-Hung Liu, Wei-Chiang Shen

**Affiliations:** Department of Plant Pathology and Microbiology, National Taiwan University, Taipei 10617, Taiwan; david0517@gmail.com

**Keywords:** *Cryptococcus neoformans*, *CRK1*, *GAT1*, bisexual mating, filament differentiation

## Abstract

The heterothallic basidiomycetous fungus *Cryptococcus neoformans* has two mating types, *MAT***a** and *MAT*α. Morphological progression of bisexual reproduction in *C. neoformans* is as follows: yeast to hyphal transition, filament extension, basidium formation, meiosis, and sporulation. *C. neoformans* Cdk-related kinase 1 (*CRK1*) is a negative regulator of bisexual mating. In this study, we characterized the morphological features of mating structures in the *crk1* mutant and determined the genetic interaction of *CRK1* in the regulatory networks of sexual differentiation. In the bilateral *crk1* mutant cross, despite shorter length of filaments than in the wild-type cross, dikaryotic filaments and other structures still remained intact during bisexual mating, but the timing of basidium formation was approximately 18 h earlier than in the cross between wild type strains. Furthermore, gene expression analyses revealed that *CRK1* modulated the expression of genes involved in the progression of hyphal elongation, basidium formation, karyogamy and meiosis. Phenotypic results showed that, although deletion of *C. neoformans CRK1* gene increased the efficiency of bisexual mating, filamentation in the *crk1* mutant was blocked by *MAT2* or *ZNF2* mutation. A bioinformatics survey predicted the *C. neoformans* GATA transcriptional factor Gat1 as a potential substrate of Crk1 kinase. Our genetic and phenotypic findings revealed that *C. neoformans* *GAT1* and *CRK1* formed a regulatory circuit to negatively regulate *MAT2* to control filamentation progression and transition during bisexual mating.

## 1. Introduction

*Cryptococcus* spp. are a group of basidiomycetous fungi that can be isolated from pigeon droppings, soil and trees and cause meningitis in humans [1,2]. Infection with two major *Cryptococcus* species, *C. neoformans* and *C. gattii*, results in cryptococcosis [3]. *C. gattii* is distributed in tropical or subtropical areas and infects immunocompetent individuals [4,5,6], whereas *C. neoformans* is the major human fungal pathogen that infects AIDS patients worldwide.

*C. neoformans* grows vegetatively as yeast cells and is a bipolar heterothallic fungus that has mating type *MAT***a** and *MAT*α cells. The life cycle of *C. neoformans* has been described [7]. Several environmental conditions, such as nitrogen starvation, room temperature, and the presence of mating pheromone, trigger bisexual differentiation and same-sex mating in *C. neoformans*. Sexual development in *C. neoformans* starts with a morphological transition from a yeast form to a filamentous hypha.

Like many basidiomycetes, *C. neoformans* undergoes sexual differentiation with four major events: cell fusion, dikaryotic filament formation, meiosis, and sporulation. When bisexual mating is initiated in *C. neoformans*, *MAT***a** and *MAT*α mating type cells secrete pheromones via the transporter Ste6 [8]. Pheromones are perceived by the pheromone receptors Ste3**a** and Ste3α, which activates the heterotrimeric G proteins (Gpa2, Gpb1 and Gpg2) and p21-activated kinase (PAK) family proteins (Ste20**a**/α) [4,9,10,11,12]. Signals are transduced to the *Cryptococcus* protein kinase 1 (Cpk1) mitogen-activated protein kinase (MAPK) signaling cascade, including the core components Ste11**a**/α, Ste7, and Cpk1 [13,14,15]. *C. neoformans MAT2* is a high-mobility–group (HMG) transcription factor acting downstream of the Cpk1 MAPK pathway and binds to the pheromone response element in the promoter region of mating specific genes to promote cell fusion [5,16]. The *C. neoformans* cell identity complex Sxi1α/Sxi2**a** is activated by Mat2 directly and then induces *CLP1* expression, which is needed for dikaryotic filament generation after cell fusion [5,17,18]. Znf2, a C2H2 zinc finger transcription factor, is also specifically required for dikaryotic filament formation but dispensable for cell fusion [16,19]. Znf2 regulates the expression of *PUM1* and *BRF1* for aerial hyphae formation and hyphal differentiation [19,20,21,22]. Additionally, *C. neoformans* Kar7 and Kar5 regulate karyogamy; Csa1, Csa2 and Ubc5 regulates meiosis, basidial maturation and sporulation [15,23,24,25].

Light can repress bisexual mating in *C. neoformans* via the Bwc1/Bwc2 complex by inhibiting the cell fusion stage and reducing dikaryotic filament generation [26,27]. Agrobacterium-mediated insertional mutagenesis was used to identify putative non-filamentation suppressors mediated by the Bwc1/Bwc2 complex [28]. *C. neoformans CRK1* gene, a *Saccharomyces cerevisiae IME2* homologue, was identified and characterized [29]. In *S. cerevisiae*, Ime2 is a meiotic-specific serine/threonine protein kinase that regulates meiosis and sporulation [30]. At the onset of meiosis, *IME2* is induced by Ime1 to coordinate the follow-up meiotic progress [31,32]. The targets of *S. cerevisiae* Ime2, such as Sic1, Rfa2, Sum1, and Cdh1, are phosphorylated by Ime2 during meiosis. Other potential downstream targets of Ime2 were predicted with the Ime2 phosphoacceptor consensus sequence R-P-X-S/T-R/P/A [33,34,35,36,37,38,39,40,41].

The homologous protein kinases of Ime2 modulate different cellular functions in different fungal model systems. *Neurospora crassa ime-2* gene plays a negative role in sexual differentiation. Mutation of *N. crassa ime-2* increases the production of fruiting bodies in the presence of nitrogen. *N. crassa vib-1*, a transcription factor, regulates protoperithecia formation in *N. crassa*, which is epistatic to *ime-2* in protoperithecia formation [42]. According to a study of the *S. cerevisiae* Ime2 consensus phosphorylation site, *N. crassa* VIB-1 contains the specific site RPRS^*60^. Evidence also indicates that VIB-1 is an IME-2 phosphorylation target in *N. crassa* [42,43]. *Ustilago maydis crk1*, an *S. cerevisiae IME2* homologous gene, is an MAPK member in the pheromone response pathway and controls morphogenesis. Recent study has found that *U. maydis* Crk1 is required for sexual development and also related to early endosome motility during fungal infection [44,45,46,47,48].

Our prior study revealed that *C. neoformans CRK1* gene is a negative regulator of bisexual mating. The *crk1* mutants show higher cell fusion efficiency and increased mating filamentation, and the timing for the formation of basidia and basidiospores was earlier than that seen in the wild-type cross. In this study, we detailed the morphological features of dikaryotic filaments in the bilateral *crk1* mutant cross and conducted genetic and gene expression studies between *C. neoformans CRK1* and *MAT2* or *ZNF2* in the bisexual mating process. We also identified that Gat1, a GATA type transcription factor, is a putative target of Crk1 and suggest that *CRK1* and *GAT1* may coordinately regulate bisexual mating in *C. neoformans.*

## 2. Materials and Methods

### 2.1. Strains, Media and Growth Conditions

*C. neoformans* serotype D strains listed in Table 1 were used. All strains were cultivated by standard media and handled according to previously published protocols and techniques [49]. Yeast-peptone-dextrose (YPD) medium was routinely used for culture maintenance at 30 °C, and V8 media incubated at 26 °C were used for bisexual mating experiments, respectively. YPD medium with 200 µg/mL hygromycin B or with 100 µg/mL nourseothricin and 200 µg/mL hygromycin B was used for selecting transformants.

### 2.2. Construction of C. neoformans mat2 and znf2 Mutant Strains

To delete *MAT2* and *ZNF2* genes in *C. neoformans MAT***a** and *MAT*α wild-type and *crk1* mutant background, we generated the *MAT2-HYG* and *ZNF2-HYG* split markers for transformation by double-joint PCR as described [52,53]. The oligonucleotides used were listed in Table S1.

To generate *MAT2-HYG* split marker fragments, the 5′-and 3′-flanking regions of *MAT2* were amplified with WC1130/WC1131 and WC1132/WC1133 primers, respectively. Then, 1.2-kb of the 5′-*HYG* cassette and 1.1 kb of the *HYG*-3′ cassette, with a ~300-bp overlapped sequence, were amplified by PCR with primer pairs WC270/WC739 and WC765/WC271, respectively, with plasmid pJAF15 as DNA template. PCR products were purified, and 1 ng of each product was used for double-joint PCR with primers WC1130/WC886 and WC1133/WC885, respectively, to generate 5′ and 3′ *MAT2-HYG* split marker fragments. The amplified fragments were separated by gel electrophoresis and purified for transformation.

To generate *ZNF2-HYG* split marker fragments, the 5′- and 3′-flanking regions of *ZNF2* were amplified with primer pairs WC1256/WC1257 and WC1258/WC1259, respectively. The 5′ and 3′ overlapped fragments of the *HYG* cassette were amplified as described previously. Double-joint PCR reactions were performed with primer pairs WC1256/WC886 and WC1259/WC885 to generate 5′ and 3′ *ZNF2-HYG* split-marker fragments, respectively, for transformation.

For transformation, 1 µg DNA of each 5′ and 3′ split-marker fragment was mixed and delivered into *C. neoformans MAT***a** and *MAT*α wild-type and *crk1* mutant strains by particle bombardment. Transformants were selected on YPD medium containing 200 µ/mL hygromycin B and YPD medium containing 200 μg/mL hygromycin B and 100 μg/mL nourseothricin, and DNA was extracted by the FPFD method for PCR screening [54]. PCR reactions were performed using primer pairs WC875/WC876 and WC879/WC880 to screen for the presence of *MAT2* and *ZNF2*. The *mat2* deletion mutants failed to amplify the 120-bp wild-type *MAT2* fragment and the *znf2* mutants failed to amplify the 130-bp wild-type *ZNF2* fragment.

### 2.3. Overexpression of C. neoformans CRK1 in the MATα crk1 Mutant

The *CRK1* overexpression construct generated previously was used to deliver into *MAT*α *crk1ura5* cells by biolistic transformation [29]. DNA was extracted from uracil prototrophic transformants by the FPFD method for PCR screening [54]. Candidate strains were further verified by real-time qRT-PCR assay.

### 2.4. Overexpression of C. neoformans MAT2 in the MATα Wild-type Strain

To overexpress the *C. neoformans MAT2* gene, PCR primers WC1734 and WC1735 were designed to amplify the 2.5-kb genomic fragment containing the *MAT2* open reading frame and downstream terminator sequence. PCR product was purified and cloned into pCR-Blunt II-TOPO vector (Invitrogen, Carlsbad, CA, USA). The positive clones were verified by sequencing and digested with BamHI and KpnI to release the *MAT2* fragment. The fragment was then ligated with pYKL8, which contains a constitutive promoter of *C. neoformans GPD1*, to generate the *MAT2* overexpression construct [27]. The construct was delivered into the *MAT*α *ura5* strain JEC43 by biolistic transformation. Uracil prototrophic transformants were chosen and screened by PCR. Candidate clones were further verified by real-time qRT-PCR assay.

### 2.5. Genetic Manipulation of C. neoformans GAT1 Gene

To delete *GAT1* in *C. neoformans* serotype D wild-type JEC20 and JEC21 strains, *MAT***a** *crk1* and *MAT*α *crk1* mutants, and *MAT***a** and *MAT*α P*_GPD1_::CRK1* strains*,* we generated *GAT1-HYG* deletion split markers by using double-joint PCR. PCR was used to amplify 5′-and 3′-flanking sequences of *GAT1* with primer pairs WC2432/WC2434 and WC2435/WC2437, respectively, with JEC21 genomic DNA. The 5′-and 3′-partial regions of the *HYG* cassette were amplified. The overlapped fragment containing *GAT1* 5′-flanking and 5′-*HYG* sequences was generated by double-joint PCR with primer pair WC2433/WC886. A similar amplification procedure with primer pair WC2436/WC885 was used to amplify the fragment containing *GAT1* 3′-flanking and *HYG*-3′ sequences. Finally, 1 µg of each split-marker fragment was mixed and delivered into *C. neoformans* serotype D strains by biolistic transformation. Transformants were selected on YPD containing 200 μg/mL hygromycin B and DNA was extracted by the FPFD method for PCR screening.

To reconstitute the wild-type *GAT1* allele back into the mutant strains, the *GAT1* locus was amplified from the JEC21 wild-type strain with primers WC2594 and WC2595. PCR fragment was purified and cloned into pCR-Blunt II-TOPO vector (Invitrogen, Carlsbad, CA, USA). The clones were digested by SacI and SpeI, and the inserted fragment was subcloned into pCH233 to generate the *GAT1* reconstitution construct. The reconstitution plasmid was biolistically transformed into the *gat1* deletion mutants. Stable transformants were selected on YPD medium containing 200 μg/mL hygromycin B and 100 μg/mL nourseothricin. The reconstituted strains were confirmed by PCR and southern blot analyses for the presence of *GAT1* and subjected to phenotypic examination.

To overexpress *GAT1*, the open reading frame and downstream terminator region were amplified from JEC21 genomic DNA by PCR with primers WC2555 and WC2556 containing NotI site at the 5′ and 3′ ends, respectively. PCR product was purified and cloned into pCR-Blunt II-TOPO vector (Invitrogen, Carlsbad, CA, USA). The clones were verified by sequencing and digested with NotI to release the *GAT1* fragment. Products were then ligated into pKHL1, which contains a constitutive promoter of *C. neoformans GPD1* in pJAF15 vector [52], to generate the *GAT1* overexpression plasmid. The construct was linearized by SacI, then introduced into the *MAT***a** and *MAT*α wild-type, and *MAT*α *crk1 + P_GPD1_::CRK1* strains by biolistic transformation. Transformants were selected on YPD medium containing 200 μg/mL hygromycin B, and DNA was extracted by the FPFD method for PCR screening [54]. Candidate clones were further verified by real-time qRT-PCR assay.

To generate the phospho-null and phospho-mimetic mutation in the predicted phosphorylation site of Gat1, site-directed mutagenesis was conducted by overlapping PCR with the mutated primer pairs WC2604/WC2605 and WC3257/WC3258, and *GAT1*-overexpression construct as a template. For phospho-null mutation, PCR primers WC2639/WC2605 and WC2604/WC1181 were used to amplify two fragments for overlapping; and for phospho-mimetic mutation, WC2639/WC3258 and WC3257/WC3258 primers were used. Overlapping PCR was conducted by using primers WC2567 and WC2573. A 2.3-kb overlapping fragment containing partial *GAT1* open reading frame with *GAT1^T1164A^* and *GAT1^T1164D^* substitution and the terminator region was cut by BamHI and SpeI, and the fragment was purified and cloned into pCR-Blunt II-TOPO vector (Invitrogen, Carlsbad, CA, USA). Correct clones were confirmed by sequencing and the fragment with substituted amino acid was released by BamHI and SpeI and subcloned into pJAF15 to respectively generate the *GAT1^T1164A^* and *GAT1^T1164D^* site-directed mutagenesis construct with the *HYG* marker. The construct was linearized by BamHI and delivered into the *MAT***a** and *MAT*α wild-type cells or *MAT***a** *crk1* and *MAT*α *crk1* mutant strains by biolistic transformation. Stable transformants were selected on YPD medium containing 200 μg/mL hygromycin B, and DNA was extracted by the FPFD method and further subjected to PCR amplification and sequencing confirmation [54].

### 2.6. Sample Preparation for Gene Expression Analyses

Strains subjected to gene expression analyses in **a**-α opposite sex mating were grown in 5 mL YPD liquid medium at 30 °C for overnight, then transferred to 45 mL liquid YPD medium and continuously grown for 22 h to reach the density at 1 × 10^8^ cells/mL. Cells were harvested, washed with sterile water and resuspended in 5 mL sterile water. An equal amount of *MAT***a** and *MAT*α cells was mixed and 10 μL of cell mixtures was spotted onto V8 agar medium and kept in the dark at 26 °C to induce mating; part of the cell suspension was centrifuged, frozen and used as a 0 h control sample for gene expression analyses. Mating cells were harvested with sterile water from the surfaces of the plates at a designated time. All harvested cells were immediately frozen in liquid nitrogen and lyophilized for RNA extraction.

Total RNA was extracted by using TRIzol reagent as described (Invitrogen). 10 μg of RNA from each sample was treated with Ambion® Turbo DNA-free™ kit (Invitrogen, Carlsbad, CA, USA) and single-strand cDNA was synthesized by using the High Capacity cDNA Reverse Transcription Kit (Applied Biosystems, Carlsbad, CA, USA ). Real-time PCR assay was performed with the Applied Biosystems StepOne real-time PCR system with KAPA SYBR FAST qPCR kits (Kapa Biosystems. Wilmington, MA, USA). *C. neoformans GPD1* gene was used to normalize relative gene expression. All samples were conducted in triplicate for gene expression assay. Two-tailed Student’s *t* test (*p*-value < 0.05) was used to compare mRNA levels between two samples. Primers used for real-time qRT-PCR experiments are listed in Supplementary Table S1.

### 2.7. Fluorescence Microscopy

Strains used for fluorescence microscopy were previously described [29]. For filament imaging, *C. neoformans* cells were spotted on SLAD agar medium containing 25 µg/mL calcofluor white and incubated at 26 °C for 13 h under dark conditions, then collected by using 1 mL sterile water. An amount of 10 µL cell suspension was spread on SLAD agar medium containing 25 µg/mL calcofluor white and covered with a coverslip. Photos were captured by using the DeltaVision system (GE Healthcare, Chicago, IL, USA) with an Olympus IX-71 inverted microscope equipped with a CoolSnap HQ2 high-resolution charged-coupled-device (CCD) camera and a 100× objective. The excitation and emission filter sets, CFP 438/24 and 470/24 and GFP/FITC 475/28 and 525/50, were used for observing calcofluor white and GFP, respectively.

## 3. Results

### 3.1. In the C. neoformans Bilateral crk1 Mutant Cross, Dikaryotic Filamentation was Altered but Nucleus Distribution Was Normal

**Figure 1:**
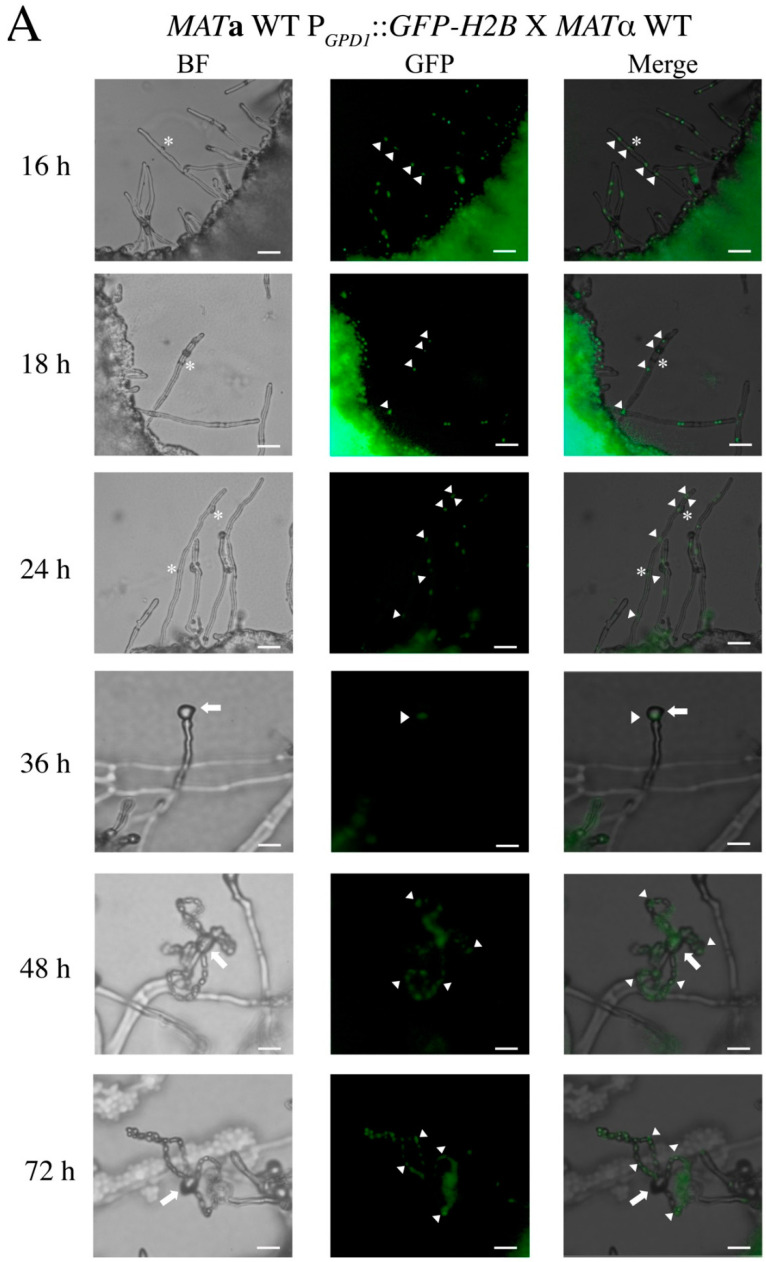

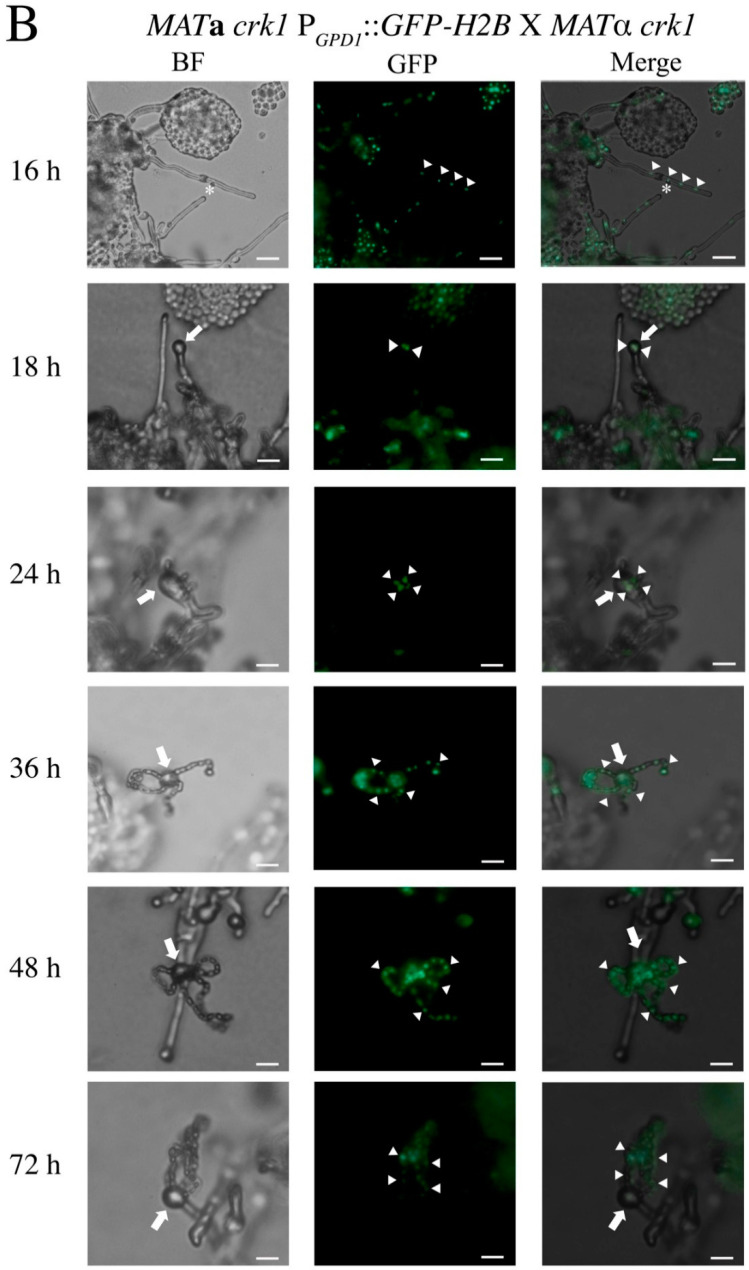
Nuclear distribution in the mating structures of the wild-type and bilateral *crk1* mutant crosses during bisexual mating process. Mating of the wild-type (**A**) and bilateral *crk1* mutant (**B**) crosses was conducted on V8 solid medium and incubated at 26 °C. Nuclei were visualized by Gfp-H2b fusion protein in the *MAT***a** wild-type and *MAT***a** *crk1* mutant cells. Bright-field (BF) and fluorescent (GFP) images of dikaryotic filaments, basidia and long chains of basidiospores were photographed at 400× magnification. Photos taken from 16 to 72 h were shown and merged photos were created with ImageJ. White star indicates clamp cell, white triangle indicates nucleus, and white arrow indicates basidia. Scale bar = 5 µm.

*C. neoformans CRK1* is a negative regulator of sexual reproduction. Sexual reproduction efficiency in the bilateral *crk1* mutant cross is increased and the production of basidiospores occurs earlier than the wild-type cross. Furthermore, dikaryotic filaments at the edges of the mating colony are shorter in the bilateral *crk1* mutant cross than the wild-type [29]. To further investigate the detailed developmental features in the bilateral *crk1* mutant cross and whether nuclear distribution in the dikaryotic filaments of the mutant cross is affected, we used the *GFP-H2B*–tagged *MAT***a** wild-type and *crk1* mutant strains, as previously described [29], to cross with the *MAT*α wild-type and *crk1* mutant strains, respectively. Bisexual mating was monitored every 2 h up to 72 h. On time-lapse observation, the length of dikaryotic filaments did not differ between the wild-type and bilateral *crk1* mutant crosses at the edge of mating colonies at 16 h (Supplementary Figure S1). Two *GFP*-tagged nuclei were observed in each hyphal cell in both the wild-type and bilateral *crk1* mutant crosses and the morphology of dikaryotic filaments was also no difference (Figure 1A,B). Furthermore, aerial hyphae differentiated to generate basidia in the bilateral *crk1* mutant cross, and two *GFP*-labeled nuclei moved to the basidium at 18 h (Figure 1B). In contrast, in the wild-type, the morphology of dikaryotic filaments remained straight, with no basidia observed (Figure 1A). In the bilateral *crk1* mutant cross, from 20 to 24 h, the growth of dikaryotic filaments at the edge of mating colonies seemed to cease and the length was generally shorter when compared to the wild-type cross (Figure S1c–e). At the same time, *GFP*-labeled nuclei in the bilateral *crk1* mutant cross underwent nuclear fusion and meiosis and generated four nuclei (Figure 1B); four long chains of basidiospores were apparently observed at 36 h and continuously until 72 h (Figure 1B). However, basidia emerged at 36 h in the wild type cross, then completed nuclear fusion and meiosis, and four long chains of basidiospores were observed at 48 h and up to 72 h (Figure 1A). Thus, during bisexual mating, the morphology of dikaryotic filaments and nuclei distribution did not differ between the wild-type and *crk1* mutant. However, basidial formation, meiosis and the production of basidiospores were earlier in the bilateral *crk1* mutant cross than the wild-type by approximately 18 h.

**Figure 2:**
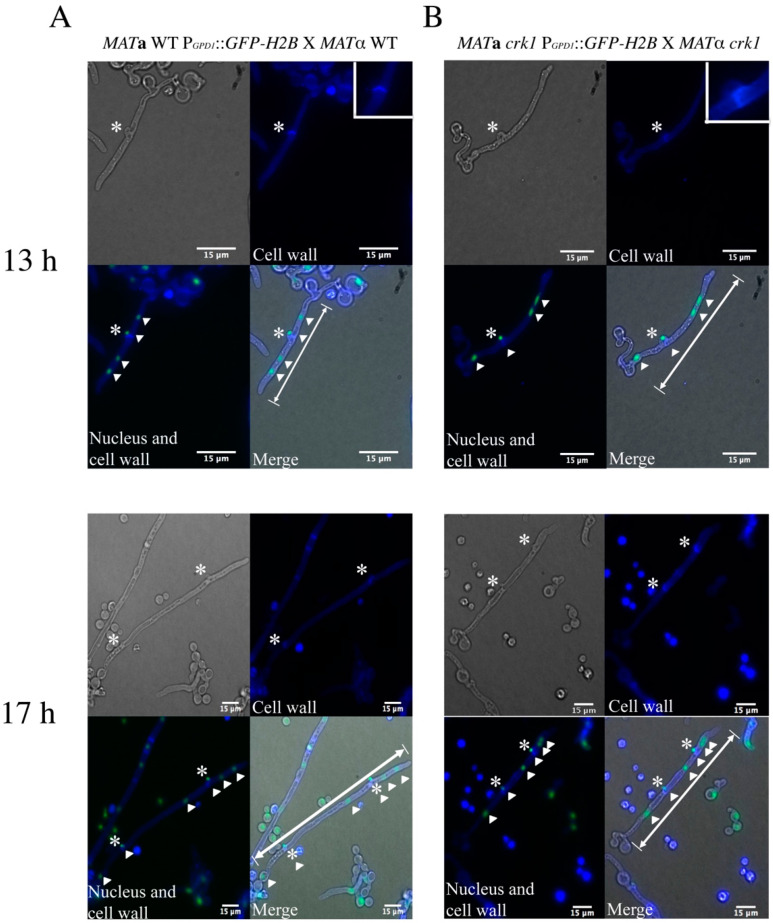
Morphology of dikaryotic filaments in the wild-type and bilateral *crk1* mutant crosses. Mating mixtures of the wild-type (**A**) and bilateral *crk1* mutant (**B**) crosses were incubated on SLAD medium containing 25 µg/mL calcofluor white and kept at 26 °C under dark condition. Nuclei were labeled by Gfp-H2b fusion protein. Cell wall was stained with calcofluor white. Dikaryotic filaments were photographed at 13 and 17 h post-incubation at 60× magnification. White star indicates clamp cell and white triangle marks nucleus. White double-headed arrow indicates the range of dikaryotic filament measurement. Scale bar = 15 µm.

Despite no change in the length of dikaryotic filaments between the wild-type and the bilateral *crk1* mutant cross before 18 h, afterward, two genotypes showed distinct lengths of dikaryotic filaments. Therefore, by using clamp cells stained with calcofluor white, we compared dikaryotic filaments between the wild-type and bilateral *crk1* mutant crosses at 13 and 17 h. The *GFP-H2B*-tagged *MAT***a** wild-type and *crk1* strains were crossed with *MAT*α wild-type and *crk1* mutant strains, respectively, on SLAD agar medium containing 25 µg/mL calcofluor white. The intact fused clamp cells emerged from dikaryotic filaments in both the wild-type and bilateral *crk1* mutant cross at 13 and 17 h (Figure 2). At 13 h, only one fused clamp was produced, and we measured the length from the tip of dikaryotic filaments to the junction of the yeast cell. After counting 120 dikaryotic filaments, which in the wild-type was a mean of 20.04 ± 4.6 µm and in the bilateral *crk1* mutant cross was 19.57 ± 3.73 µm. Then, at 17 h, the extended and straight dikaryotic filaments generated two fused clamp cells in both the wild-type and bilateral *crk1* mutant crosses. However, the length of dikaryotic filaments differed: the mean length in the wild-type was 80.1 ± 7.22 µm and in the bilateral *crk1* mutant cross was 38 ± 6.47 µm. Collectively, our detailed cytological characterization revealed that short dikaryotic filament phenotype exhibited by the *crk1* mutant was due to reduced length of the hyphal segment after 13 h incubation but nuclear distribution and behavior were unaltered inside the mating structures, suggesting that progression of dikaryotic filamentation and further transition to basidium might be mediated by *C. neoformans* Crk1.

### 3.2. C. neoformans CRK1 Repressed Cell Fusion, Filamentation, Karyogamy and Meiotic Gene Expression during Bisexual Mating

**Figure 3:**
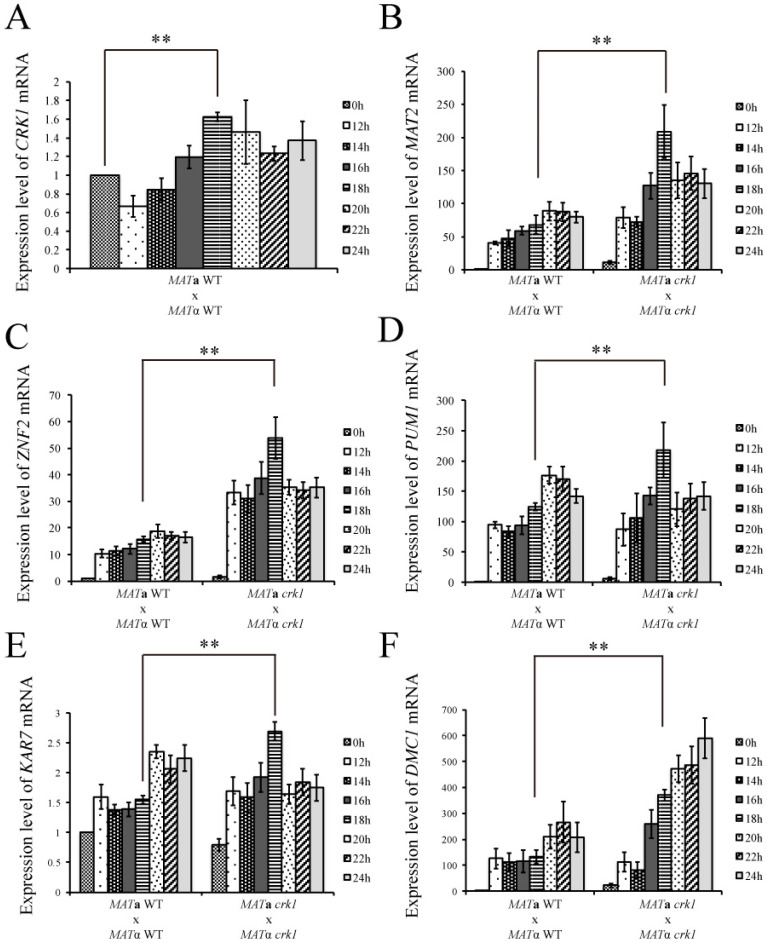
The expression of mating-, hyphal extension-and sporulation-related genes was elevated at 18 h in the bilateral *crk1* mutant cross. Bilateral crosses involved the *MAT***a** and *MAT*α wild-type and *crk1* mutants were conducted on V8 agar plates and incubated at 26 °C in the dark. Samples were collected at 0, 12, 14, 16, 18, 20, 22 and 24 h post-incubation. The expression of (**A**) *CRK1*, (**B**) *MAT2*, (**C**) *ZNF2,* (**D**) *PUM1,* (**E**) *KAR7,* and (**F**) *DMC1* was examined by real-time qRT-PCR analysis. Triplicate reactions for each sample were conducted. Error bar represents the standard deviation from the mean of three replicates. The results were normalized to *C. neoformans GPD1* expression. (** indicates *p* < 0.005).

The Cpk1 MAPK cascade is one of the key signaling pathways that govern pheromone transduction, cell fusion, and dikaryotic filament emergence in *C. neoformans*. To determine the effect of *C. neoformans CRK1* mutation on the expression of the genes involved in the Cpk1 MAPK signaling pathway, dikaryotic filamentation, and fruiting structure development, we conducted gene expression analyses in the wild-type and *crk1* mutant. Because we determined the morphological features of the wild-type and *crk1* mutant in dikaryotic filamentation from 16 h to 72 h during bisexual mating, mating samples at 0, 12, 14, 16, 18, 20, 22, and 24 h post-incubation on V8 medium were selected for gene expression study and analyzed by real-time qRT-PCR. In the wild-type, *CRK1* expression was maintained at low levels from 12 to 24 h, and the lowest mean mRNA expression for *CRK1* was 0.66 ± 0.11-fold at 12 h but gradually increased and peaked at 1.62 ± 0.04-fold at 18 h (Figure 3A).

*C. neoformans MAT2* gene is a major transcription factor that regulates pheromone production and stimulates cell fusion [16]. We next examined the *MAT2* expression pattern in the wild-type and bilateral *crk1* mutant crosses. The *MAT2* expression was higher in the bilateral *crk1* mutant cross than the wild-type from 12 to 24 h. The mean level of *MAT2* in the wild-type was 48.49 ± 3.9-fold at 12 h, then peaked at 88.58 ± 13.7-fold at 20 h. However, the mean mRNA level of *MAT2* in the bilateral *crk1* mutant cross was 79.03 ± 16.02-fold at 12 h and the peak expression was 208 ± 39.69-fold at 18 h. After 18 h, the mRNA level of *MAT2* decreased (Figure 3B).

*C. neoformans ZNF2* gene is the major transcription factor that regulates filament development and also induces *PUM1* to generate aerial hyphae during bisexual mating [21]. *ZNF2* expression was upregulated in the bilateral *crk1* mutant cross as compared with the wild-type, with peak level at 18 h (Figure 3C). In contrast, the level of *ZNF2* peaked at 18.86 ± 2.52-fold at 20 h in the wild-type but remained significantly lower than that for the bilateral *crk1* mutant cross (Figure 3C). The wild-type and bilateral *crk1* mutant cross did not differ in the level of *PUM1* at 12 h and 14 h (Figure 3D). At 16 and 18 h, *PUM1* expression increased significantly in the bilateral *crk1* mutant cross and peaked at 18 h. The peak expression for *PUM1* in the wild-type was at 20 h (Figure 3D). Furthermore, both *ZNF2* and *PUM1* expression was downregulated in the bilateral *crk1* mutant cross after 18 h (Figure 3C,D).

We also assessed the transcript levels of karyogamy-and meiotic-related genes *KAR7* and *DMC1*. In the wild-type, the level of *KAR7* was stable from 12 to 18 h, then increased at 20 h. In the bilateral *crk1* mutant cross, the expression level of *KAR7* was elevated earlier than the wild-type at 16 and 18 h but decreased from 20 to 24 h (Figure 3E). The expression of *DMC1* in the wild-type initially showed no significant changes from 12 to 18 h but increased at 20 h and peaked at 22 h (Figure 3F). In the bilateral *crk1* mutant cross, *DMC1* expression was similar to that of the wild- type at 12 and 14 h. However, the expression markedly increased at 16 h and continuously increased until 24 h (Figure 3F). Hence, our gene expression results showed that the mutation of *CRK1* resulted in upregulation of cell fusion-, filamentation-, karyogamy-and meiotic-related genes at about 16 to 18 h during bisexual mating, indicating *C. neoformans CRK1* played important roles in regulating various bisexual differentiation processes.

### 3.3. CRK1 Expression Level was Decreased in the Bilateral mat2 Mutant Cross

**Figure 4:**
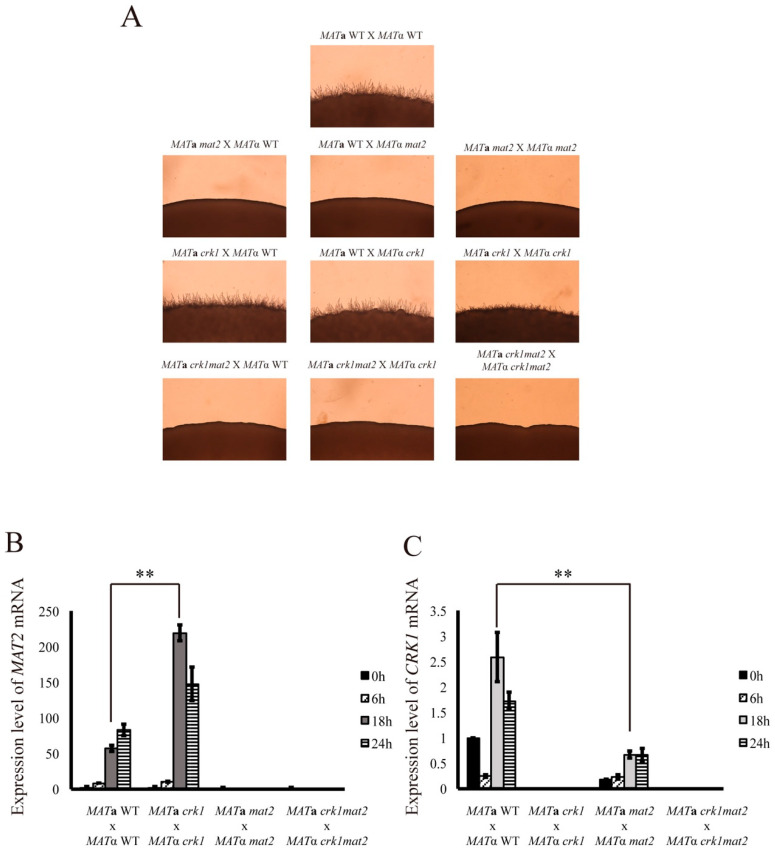
Sexual differentiation in the *crk1* mutant was blocked by the mutation of *MAT*2 and *CRK1* expression was reduced in the bilateral *mat2* mutant cross. (**A**) *C. neoformans MAT***a** and *MAT*α strains were crossed as indicated. Mating was conducted on V8 agar plates and incubated at 26 °C in the dark. Photos were taken 24 h post-incubation at 100× magnification. Bilateral crosses involved the *MAT***a** and *MAT*α wild-type and *crk1*, *mat2*, and *mat2crk1* double mutants were conducted. Samples were collected at 0, 6, 18 and 24 h post-incubation and subjected to RNA extraction. The expression of *MAT2* (**B**) and *CRK1* (**C**) during bisexual mating was examined by real-time qRT-PCR analysis. Triplicate reactions for each sample were conducted. Error bar represents the standard deviation from the mean of three replicates. The results were normalized to *C. neoformans GPD1* expression. (** indicates *p* < 0.005).

In this research, we confirm that *MAT2* expression was increased in the bilateral *crk1* mutant cross. Our previous study also showed that *CRK1* overexpression downregulates the transcript level of *MAT2* during bisexual mating [29]. To further assess the relation between *CRK1* and *MAT2*, we generated the *crk1mat2* double mutant for phenotypic and gene expression analyses. Unilateral and bilateral crosses of the *mat2*, *crk1*, and *crk1mat2* mutant strains were examined on V8 medium for 24 h. Dikaryotic filaments were observed in the bilateral *crk1* mutant cross but were blocked under *mat2* deletion (Figure 4A). Both unilateral and bilateral *crk1mat2* double mutants did not produce dikaryotic filaments, even when the *crk1mat2* double mutant was crossed with the *crk1* mutant strain. Furthermore, we examined *CRK1* and *MAT2* expression in the wild-type and *crk1*, *mat2* and *crk1mat2* double mutant during bisexual mating on V8 medium at 0, 6, 18 and 24 h. *MAT2* mRNA level was not detected in the bilateral *mat2* or *crk1mat2* double mutant cross, and *CRK1* was not detected in the bilateral *crk1* or *crk1mat2* double mutant cross. In the bilateral *crk1* mutant cross, the level of *MAT2* was similar to that in the wild-type at 6 h but then became higher than the wild-type starting at 12 h (Figure 4B), as also demonstrated in Figure 3B. Interestingly, the level of *CRK1* was downregulated in the bilateral *mat2* mutant cross (Figure 4C).

**Figure 5:**
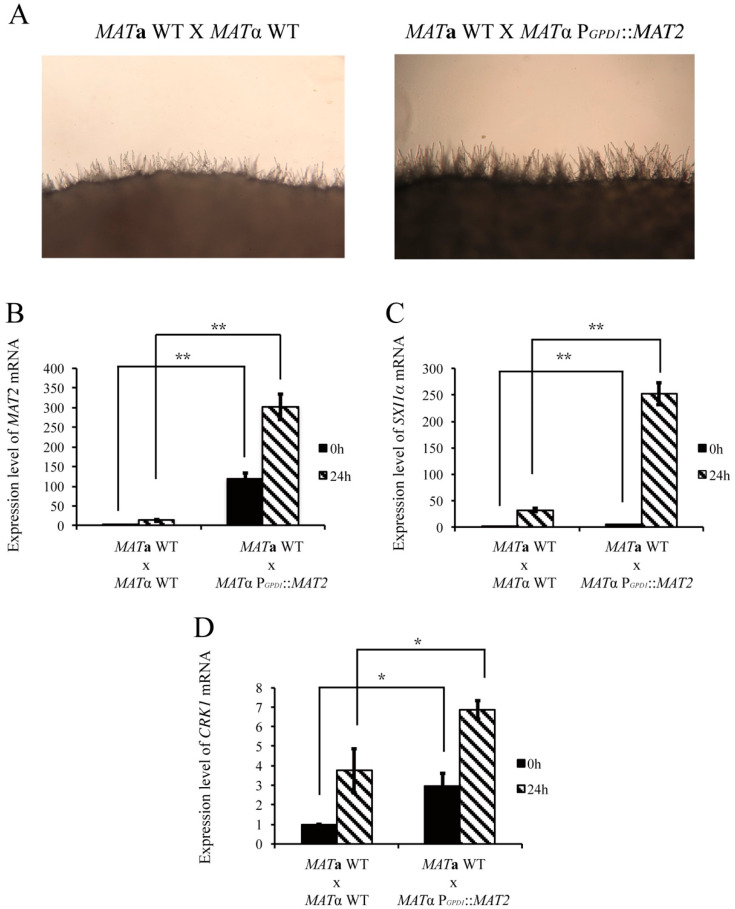
Overexpression of *MAT2* increased bisexual filamentation and upregulated *CRK1* expression. (**A**) Crosses involved *C. neoformans MAT***a** and *MAT*α wild-type and *MAT***a** wild-type and *MAT*α P*_GPD1_*::*MAT2* strains were conducted on V8 agar plates incubated at 26 °C in the dark. Photos were taken 24 h post-incubation at 100× magnification. The same crosses were also subjected to gene expression studies and samples were collected at 0 and 24 h post-incubation. The expression of *MAT2* (**B**), *SXI1*α (**C**), and *CRK1* (**D**) during mating was determined by real-time qRT-PCR analysis. Triplicate reactions for each sample were conducted. Error bar represents the standard deviation from the mean of three replicates. The results were normalized to *C. neoformans GPD1* expression. (** indicates *p* < 0.005; * indicates *p* < 0.05).

Because the mRNA level of *CRK1* was decreased in the bilateral *mat2* mutant cross, we further assessed the effect of *MAT2* overexpression on the *CRK1* level during bisexual mating. *MAT2* overexpression was constructed in the *C. neoformans MAT*α strain, and bisexual mating was conducted on V8 medium for 24 h. Profuse filaments were observed around the edge of mating colonies in the *MAT*α P*_GPD1_*::*MAT2* strain cross in comparison with the wild-type cross (Figure 5A). The mRNA level of *MAT2* was greatly elevated in the *MAT*α P*_GPD1_*::*MAT2* strain (Figure 5B). The expression of *SXI1*α, a downstream target of *MAT2* [5], was also increased markedly at 0 and 24 h and was significantly higher than in the wild-type (Figure 5C). The mRNA level of *CRK1* at 0 and 24 h was higher than in the wild-type (Figure 5D). Thus, the deletion of *MAT2* blocked dikaryotic filamentation of the *crk1* mutants during the bisexual mating process. However, *C. neoformans MAT2* likely regulated *CRK1* at the transcription level.

### 3.4. Deletion of C. neoformans ZNF2 Resulted in Non-Filamentation in the crk1 Mutant

*C. neoformans ZNF2* is a positive regulator for filament generation; deletion of *ZNF2* abolished the formation of dikaryotic filaments [16,19]. Previously, the expression of *ZNF2* was found negatively regulated by *CRK1* during bisexual mating. To assess whether *crk1* deletion could restore filamentation in the *znf2* mutant during bisexual differentiation, we generated the *crk1znf2* double mutant to examine the bisexual mating phenotype and compared with the wild-type, *crk1* and *znf2* crosses. As previously described, abundant filaments were seen in the *crk1* cross, and filamentation was blocked in the bilateral *znf2* mutant cross. Similarly, no dikaryotic filaments were observed in the bilateral *crk1znf2* mutant cross and *ZNF2* deletion blocked the filamentation phenotype of *znf2* mutant (Figure S2A).

To further determine the relation between *CRK1* and *ZNF2*, we examined the expression of *CRK1* in the wild-type and *znf2* deletion strains during bisexual mating on V8 medium for 6, 18 and 24 h. *MF*α pheromone gene is a positive indicator for bisexual mating process. The *MF*α expression was upregulated, peaked at 18 h in the *znf2* cross and then decreased at 24 h. In contrast, the expression of *MF*α was lower in the wild-type than the *znf2* cross (Supplementary Figure S2B) as previously described [16]. The mRNA levels of *CRK1* did not differ between the wild-type and *znf2* cross (Supplementary Figure S2C). Our results suggested that *CRK1* was not regulated by *ZNF2*, and deletion of the negative regulator *CRK1* did not restore filamentation in the *znf2* mutant background.

### 3.5. Gat1 Contains the Predicted Crk1 Consensus Phosphorylation Site

*C. neoformans CRK1* is a homologue of *S. cerevisiae IME2* gene. The *IME2* phosphoacceptor consensus sequence R-P-X-S/T-R/P/A was characterized and predicted in several fungal species, including *N. crassa* and *C. neoformans* [35,42,43]. To predict the putative phosphorylation targets of *C. neoformans* Crk1, we used a bioinformatic strategy to search the *C. neoformans* JEC21 database for proteins that contain the modified *S. cerevisiae* Ime2 phosphorylation consensus sequence R-P-X-S/T-R/P/A. Overall, 651 putative phosphorylation targets of Crk1 were predicted from the *C. neoformans* JEC21 genome and proteins containing DNA binding motif were further selected. From the *C. neoformans* JEC21 transcription factor database, 22 genes predicted as transcription factors were further identified, and these homologous genes in *C. neoformans* H99 were identified (Supplementary Table S2). Nitrogen starvation is one of the conditions that induces bisexual mating in *C. neoformans* [4]. The GATA type transcription activator Gat1 was predicted as a putative Crk1 phosphorylated substrate in our bioinformatic survey. *C. neoformans GAT1* is homologous to *N. crassa nit2* and *Schizosaccharomyces pombe gaf1*, the regulators of nitrogen metabolism [55,56,57]. *C. neoformans* Gat1 contains the GATA zinc-finger domain at the C-terminus (1186 to 1230 aa), and the putative *IME2* phosphorylation consensus sequence was predicted at RPGT^*1164^. Previous studies indicated that *GAT1* has a negative function for bisexual mating in *C. neoformans* H99 [58,59]. We further tested its function for bisexual differentiation in *C. neoformans* JEC21 and a genetic association with *C. neoformans CRK1*.

### 3.6. Deletion of GAT1 Enhanced Pheromone Expression and Increased Aerial Hyphae Formation

**Figure 6:**
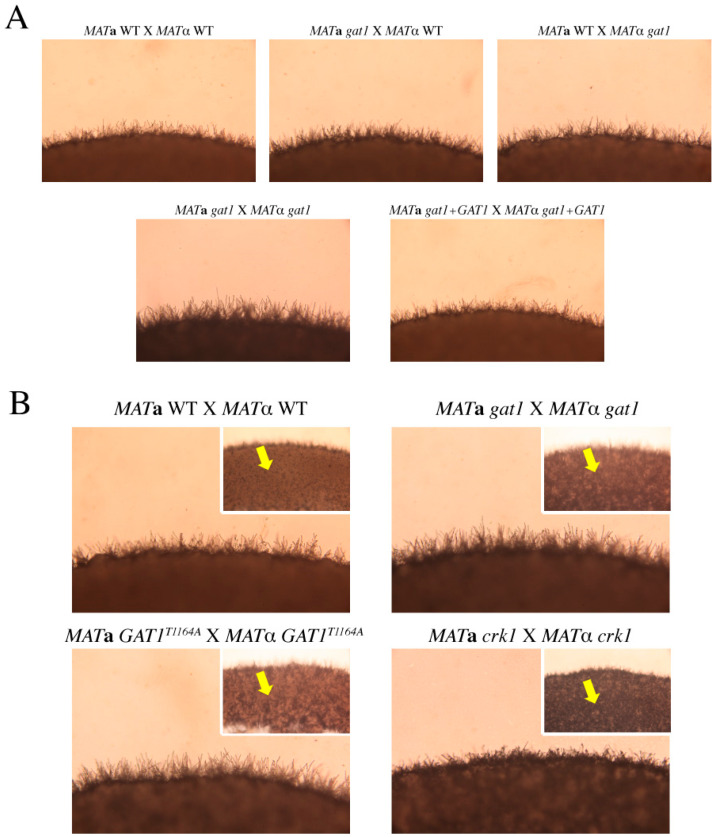
Dikaryotic filamentation and aerial hyphae formation were enhanced in the bilateral *gat1* mutant and *GAT1*^T1164A^ mutant crosses. (**A**) *C. neoformans MAT***a** and *MAT*α *gat1* mutant and overexpression strains were crossed as indicated and compared to the wild type cross. (**B**) *C. neoformans MAT***a** and *MAT*α *GAT1*^T1164A^ bilateral mutant cross was performed and compared to the crosses as indicated. Small photos reveal the density of aerial hyphae. Yellow arrow indicates aerial hyphae. Mating was conducted on V8 agar plates and incubated at 26 °C in the dark. Photos were taken 24 h post-incubation at 100× magnification.

To dissect the function of *GAT1* in bisexual mating, we generated *gat1* deletion strains by double-joint PCR in both *MAT***a** and *MAT*α wild-type strains. Complementation of the mutant strains with the wild-type *GAT1* gene was also constructed. Bisexual mating assay for the unilateral and bilateral *gat1* mutant crosses was conducted on V8 medium in the dark. After 24-h incubation, the abundance of dikaryotic filaments around the edge of mating mixture was slightly enhanced in the unilateral *gat1* cross and more profuse filaments were obviously seen in the bilateral *gat1* cross as compared with the wild-type cross (Figure 6A). Increased filamentation was similarly observed in the bilateral *crk1* mutant cross, however, filaments were longer in the bilateral *gat1* mutant cross than bilateral *crk1* mutant cross. Furthermore, profuse aerial hyphae were generated in the bilateral *gat1* mutant cross on the surface of mating mixture, which was similar to the bilateral *crk1* mutant cross (Figure 6B).

**Figure 7:**
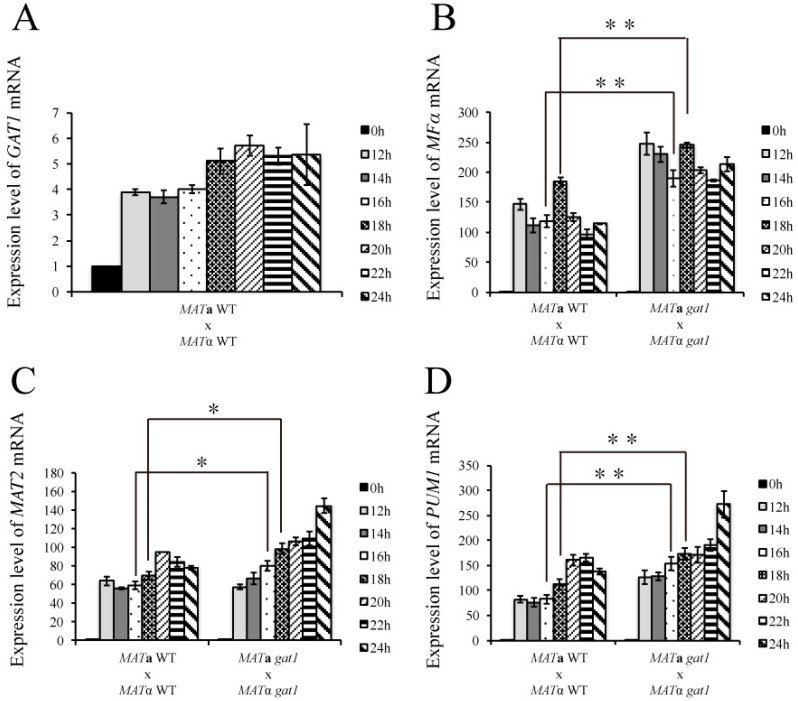
The expression of mating- and hyphal extension-related genes was increased in the bilateral *gat1* mutant cross. Bilateral crosses involved the *MAT***a** and *MAT*α wild-type and *gat1* mutants were conducted on V8 agar plates and incubated at 26 °C in the dark. Samples were collected at 0, 12, 14, 16, 18, 20, 22 and 24 h post-incubation. The expression of *GAT1* (**A**), *MF*α (**B**), *MAT2* (**C**), and *PUM1* (**D**) was examined by real-time qRT-PCR analysis. Error bar represents the standard deviation from the mean of three replicates.Triplicate reactions for each sample were conducted. The results were normalized to *C. neoformans GPD1* expression. (** indicates *p* < 0.005; * indicates *p* < 0.05).

To investigate how *C. neoformans GAT1* gene is involved in the signaling networks of mating process, the wild-type and *gat1* bilateral mutant crosses were conducted on V8 medium, and mating cells were harvested at 12, 14, 16, 18, 20, 22, and 24 h for gene expression studies by real-time qRT-PCR. The expression of *GAT1* in the wild-type cross was 3.88 ± 0.12-fold at 12 h and reached to the highest expression level at 20 h (Figure 7A). The mRNA level of *MF*α in the wild-type cross was elevated during mating process, peaked at 18 h and then decreased afterwards (Figure 7B). *MF*α expression was increased greatly since 12 h in the bilateral *gat1* mutant cross and maintained at high levels till 24 h. In addition, we further analyzed the mRNA levels of *MAT2* and *PUM1* to correlate the pheromone expression and aerial hyphae formation. Interestingly, the expression levels of *MAT2* between the wild-type and bilateral *gat1* mutant cross were similar at 12 and 14 h, but were increased since 16 h in the bilateral *gat1* mutant cross (Figure 7C). The expression of *PUM1* was generally increased in the bilateral *gat1* mutant cross as compared with the wild-type cross and significantly up-regulated at 24 h (Figure 7D). Thus, according to phenotypic and expression analysis, *C. neoformans GAT1* also played a negative role in the regulation of mating-related genes and deletion of *GAT1* resulted in abundant aerial hyphae formation.

### 3.7. The C. neoformans crk1gat1 Mutants Exhibited Similar Phenotypes as the crk1 Mutant in Bisexual Mating

**Figure 8:**
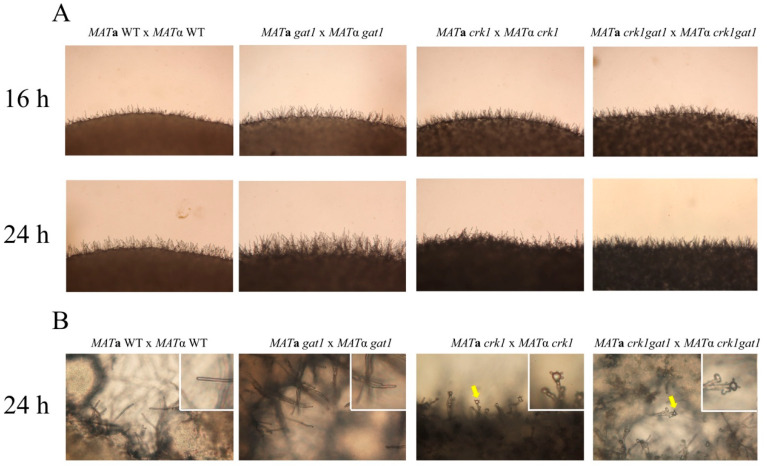
The *crk1* and *crk1gat1* mutants were phenotypically identical in bisexual mating. *C. neoformans MAT***a** and *MAT*α *gat1*, *crk1*, and *crk1gat1* mutant strains were crossed on V8 agar plates at 26 °C in the dark and compared to the wild type strains. The edges of mating mixtures were photographed 16 h and 24 h post-incubation at 100× magnification. Photos of mating filaments were also recorded at 400× magnification. Small photos illustrate the tip of dikaryotic filament and yellow arrow indicates basidium.

As we suggested before that Crk1 may phosphorylate Gat1 to regulate bisexual mating in *C. neoformans*, we further generated the *crk1gat1* mutants to further dissect the genetic relationship between *CRK1* and *GAT1* in bisexual mating. The bilateral *crk1gat1* mutant cross were conducted on V8 medium in the dark. After 24 h incubation, short dikaryotic filaments at the edge of mating colonies were observed as the bilateral *crk1* mutant cross and basidia also emerged both in the bilateral *crk1* and *crk1gat1* mutant cross (Figure 8A,B). We also further examined the expression levels of *MF*α and *MAT2* genes. The wild-type, bilateral *gat1* mutant, *crk1* mutant and *crk1gat1* mutant crosses were incubated on V8 medium and mating cells were harvested at 18 and 24 h for real-time qRT-PCR analyses. As shown in Supplementary Figure S3A,B, *MF*α and *MAT2* expression levels in the bilateral *crk1* and *crk1gat1* mutant crosses were higher than those in the wild-type and *gat1* mutant crosses. The transcript levels of *MF*α in the bilateral *crk1gat1* mutant cross were higher than those of the *crk1* mutant cross both at 18 and 24 h. The transcript levels of *MAT2* at 18 h in the bilateral *crk1* and *crk1gat1* mutant crosses were higher than those of the wild-type and bilateral *crk1* mutant crosses. *MAT2* expression level in the bilateral *crk1gat1* mutant cross was also higher in comparison to the bilateral *crk1* mutant cross. The *C. neoformans CSA1* gene is a key regulator for basidial formation [25], and *DMC1* is responsible for meiotic process. As expected, these two genes were expressed at the similar levels in the bilateral *crk1* and *crk1gat1* mutant crosses at 18 and 24 h, but significantly higher than the wild-type and bilateral *gat1* mutant crosses (Supplementary Figure S3C,D). Altogether, *GAT1* and *CRK1* both regulated the expression of mating-related genes and they may function in the same and independent signaling pathways.

### 3.8. The Predicted IME2 Consensus Phosphorylation Site Was Important for the Function of Gat1 in Bisexual Mating

*C. neoformans* Gat1 was predicted as a putative phosphorylation substrate of Crk1 and contained the Ime2 consensus phosphorylation site RPGT^*1164^. To test whether this site was important for Gat1 function to regulate bisexual mating, we conducted site-directed mutagenesis to create a *gat1* phospho-null allele with the Ime2 consensus phosphorylation site RPGT^*1164^ mutated to RPGA^*1164^, and also a *gat1* phosphomimetic allele with RPGD^*1164^. The bilateral *GAT1*^T1164A^ or *GAT1*^T1164D^ mutant crosses were conducted on V8 medium for 24 h and compared with the wild-type and bilateral *gat1* mutant crosses. In the bilateral *GAT1*^T1164A^ cross, the abundance of dikaryotic filaments and aerial hyphae were similar to that in the bilateral *gat1* mutant cross (Figure 6B). For gene expression, *GAT1* expression was not detected in the bilateral *gat1* mutant cross. However, the expression levels at 24 h were similarly up-regulated in the wild-type, bilateral *crk1* mutant cross and bilateral *GAT1*^T1164A^ cross (Supplementary Figure S9A). *MF*α expression was upregulated in the bilateral *crk1*, *gat1, and GAT1*^T1164A^ mutant crosses in comparison to the wild-type cross (Supplementary Figure S9B).

**Figure 9:**
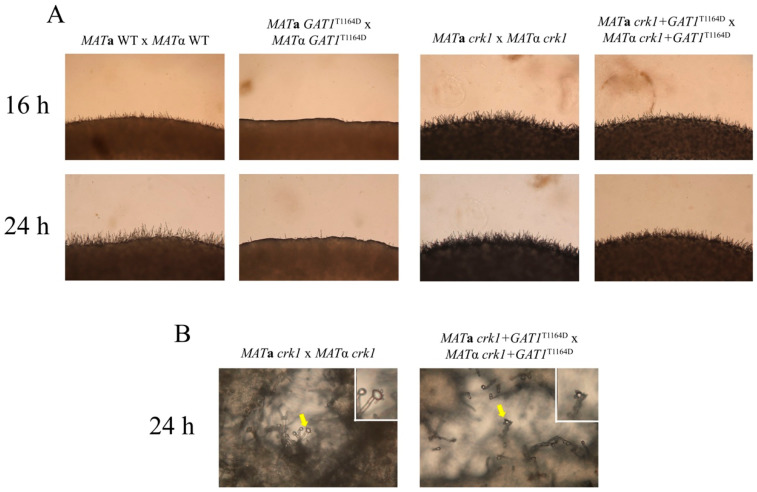
The *GAT1*^T1164D^ phospho-mimetic active allele dramatically repressed dikaryotic filamentation in the wild-type cross but showed slight effects on mating under *crk1* mutant background. (**A**) *C. neoformans MAT***a** and *MAT*α strains were crossed as indicated. Mating was conducted on V8 agar plates and incubated at 26 °C in the dark. Photos were taken 16 h and 24 h post-incubation at 100× magnification. (**B**) Small photos demonstrate the basidia in the *crk1* and *crk1GAT1*^T1164D^ bilateral mutant crosses respectively. Yellow arrow indicates basidium. Photos were taken 24 h post-incubation at 400× magnification.

**Figure 10:**
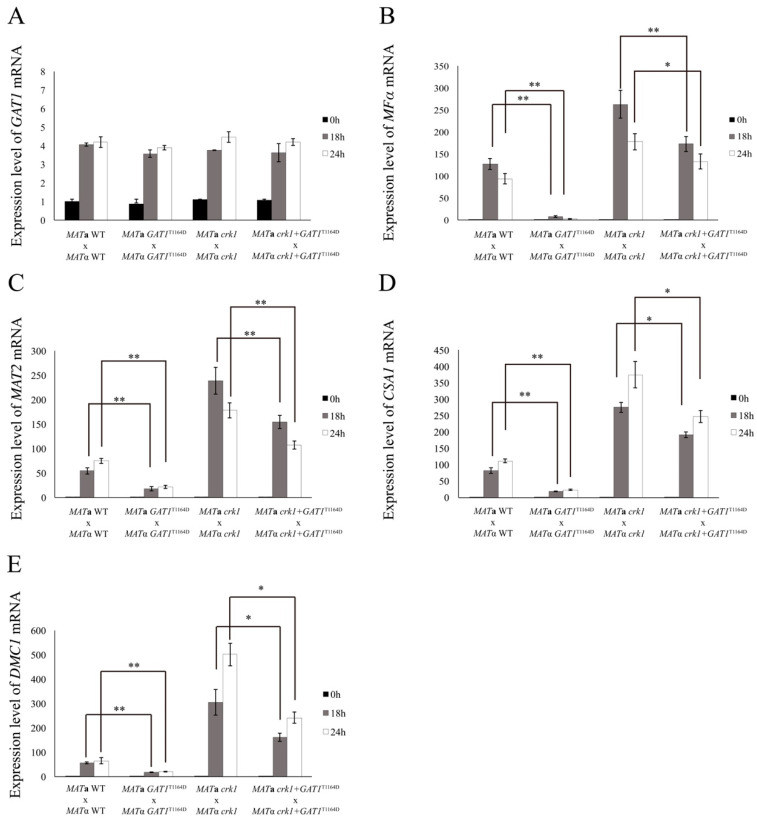
The expression of mating-related genes was down-regulated by the *GAT1* phospho-mimetic allele. *C. neoformans MAT***a** and *MAT*α strains were crossed as indicated. Mating was conducted on V8 agar plates and incubated at 26 °C in the dark. Samples were collected at 0, 18, and 24 h post-incubation. The expression of *GAT1* (**A**), *MF*α (**B**), *MAT2* (**C**), *CSA1* (**D**), and *DMC1* (**E**) was examined by real-time qRT-PCR analysis. Triplicate reactions for each sample were conducted. Error bar represents the standard deviation from the mean of three replicates. The results were normalized to *C. neoformans GPD1* expression. (** indicates *p* < 0.005; * indicates *p* < 0.05).

On the other hand, the *MAT***a** and *MAT*α wild-type strains or *crk1* mutants containing the *gat1* phosphomimetic allele were used to conduct bisexual mating on V8 medium and filamentation was assessed and compared with the wild-type and bilateral *crk1* mutant crosses at 16 h and 24 h. In the bilateral *GAT1^T1164D^* mutant cross, dikaryotic filaments were dramatically repressed at 16 h and 24 h as compared to the wild-type cross (Figure 9A). Gene expression studies revealed that the *GAT1* mRNA levels at 18 h and 24 h in the cross of *GAT1* phosphomimetic strains showed no significant difference when compared to the wild-type and bilateral *crk1* mutant cross (Figure 10A). In contrast, the transcript levels of *MF*α and *MAT2* were all significantly reduced (Figure 10B,C). In the bilateral *crk1*+ *GAT1*^T1164D^ mutant cross, dikaryotic filaments at the edge of the mating mixture were less abundant as compared to the bilateral *crk1* mutant cross both at 16 h and 24 h, but still more than wild-type cross (Figure 9A). Interestingly, the detailed features in the *crk1*+ *GAT1*^T1164D^ mutant cross mimicked those in the *crk1* cross. Dikaryotic filaments were shorter and basidia could be seen at 24 h; however, the amount of basidia in the *crk1*+ *GAT1*^T1164D^ mutant cross was less than the bilateral *crk1* cross (Figure 9B). Furthermore, the *GAT1* mRNA levels at 18 h and 24 h in the cross of *crk1*+ *GAT1*^T1164D^ mutants were also comparable to the levels in the wild-type and bilateral *crk1* mutant cross (Figure 10A). The expression levels of *MF*α and *MAT2* in the *crk1*+ *GAT1*^T1164D^ mutants cross were reduced as compared to the *crk1* mutants cross, but still maintained at high levels in comparison to the wild-type cross (Figure 10B,C). The expression levels of *CSA1* for basidial formation and *DMC1* for meiosis were similarly down-regulated in the *crk1*+ *GAT1*^T1164D^ mutants cross when compared to the *crk1* mutant cross (Figure 10D,E). Taken together, our results demonstrated that the Ime2 consensus phosphorylation site of Gat1 was essential for the proper regulatory function of Gat1 in bisexual mating.

### 3.9. Deletion of GAT1 Partially Restored Dikaryotic Filamentation of CRK1 Overexpression Strain

Previous study showed that *CRK1* overexpression represses the formation of dikaryotic filament and also reduces the expression levels of the mating-related genes [29]. In this study, we revealed that *GAT1* may be one of the downstream targets of *CRK1*. We hypothesized that deletion of *GAT1* under the *CRK1* overexpression background may restore the production of dikaryotic filament. To address this question, we deleted the *GAT1* gene in the *MAT***a** and *MAT*α *CRK1* overexpression strains and bisexual mating was then conducted on V8 medium. After 24 h incubation, dikaryotic filamentation was repressed dramatically in the bilateral cross of *CRK1* overexpression strains (Figure S7). As expected, filamentation was partially restored in the bilateral cross involved the *MAT***a** and *MAT*α *CRK1* overexpression + *gat1* mutants. However, the level of filamentation was much less than those observed in the wild-type and bilateral *gat1* mutant cross (Supplementary Figure S7).

Gene expression analysis was further performed to confirm the phenotypic observation. Mating samples were harvested at 18 and 24 h and the transcript levels of *MF*α and *MAT2* were examined. At 18 and 24 h, the *MF*α expression levels, 56 ± 12.93 and 39.8 ± 8.57 fold respectively, in the bilateral *CRK1* overexpression + *gat1* mutant cross were higher than those in the bilateral *CRK1* overexpression cross (Figure S8A). The *MAT2* mRNA levels, 21.6 ± 0.97 fold at 18 h and 28.9 ± 5.59 fold at 24 h, in the bilateral *CRK1* overexpression + *gat1* mutant cross were also higher than those in the bilateral *CRK1* overexpression cross (Figure S8B). However, the expression levels of these two mating-related genes in the bilateral *CRK1* overexpression + *gat1* mutant cross were not as high as the levels seen in the wild-type and bilateral *gat1* mutant crosses (Supplementary Figure S8A,B). Taken together, our results demonstrated that deletion of *GAT1* can only partially restore the repression of dikaryotic filamentation and gene expression conferred by *CRK1* overexpression, suggesting that Gat1 may not be the only downstream target of Crk1 to regulate bisexual mating.

### 3.10. C. neoformans CRK1 Coordinated with GAT1 to Repress Bisexual Differentiation

According to our previous results, deletion of *C. neoformans GAT1* enhanced the formation of dikaryotic filaments and increased the mRNA level of *MF*α pheromone gene. To examine the effect of *GAT1* overexpression on bisexual differentiation, we expressed *GAT1* under the *C. neoformans GPD1* constitutive promoter in the JEC20 and JEC21 strains. The *GAT1* transcript level was markedly increased in the bisexual cross of *GAT1* overexpression strains (Supplementary Figure S5A). However, no discernible phenotype was observed when compared to the wild-type cross (Supplementary Figure S4), and *MF*α expression level was similar to that in the wild-type cross (Supplementary Figure S5B). Hence, the overexpression of *GAT1* possibly had no effect on bisexual mating.

Previous studies demonstrated that the degree of bisexual mating inhibition depends on *CRK1* expression [30]. To examine whether overexpression of *GAT1* required the proper level of *CRK1* to exhibit the filament inhibition phenotype, we tried to elevate the expression levels of *CRK1* and *GAT1* under the *GPD1* promoter in the *MAT*α *crk1* strain. We transformed P*_GPD1_*::*CRK1* plasmid into the *MAT*α *crk1* mutant and selected the transformants which showed filamentation phenotypes like the wild-type strains when crossed with the *MAT***a** wild-type or *crk1* mutant strain. We then further transformed P*_GPD1_*::*GAT1* construct into the *MAT*α *crk1* + P*_GPD1_*::*CRK1* strain to generate the *MAT*α *crk1* + P*_GPD1_*::*CRK1* + P*_GPD1_*::*GAT1* strain. Bisexual mating was conducted on V8 agar medium for 24 h. Dikaryotic filamentation of the *MAT*α *crk1* + P*_GPD1_*::*CRK1* was similar to that in the wild-type and *MAT*α *crk1* unilateral mutant cross (Supplementary Figure S6A–C), and the mRNA levels of *GAT1* and *MF*α in the *MAT*α *crk1* + P*_GPD1_*::*CRK1* unilateral cross were similar and those in the wild-type cross (Supplementary Figure S9A,B). Interestingly, dikaryotic filamentation was greatly decreased in the *MAT*α *crk1* + P*_GPD1_*::*CRK1* + P*_GPD1_*::*GAT1* strain (Supplementary Figure S6D), and the expression level of *MF*α was significantly decreased in the *MAT*α *crk1* + P*_GPD1_*::*CRK1* + P*_GPD1_*::*GAT1* cross (Supplementary Figure S9B). Thus, *C. neoformans CRK1* likely coordinated with *GAT1* to negatively regulate the process of bisexual mating.

## 4. Discussion

The evolutionarily flexible functions of fungal Ime2/Crk1 kinases reported in several fungal species play an important role in sexual differentiation and also regulate programmed cell death, endosome motility, extracellular protease activity, mycotoxin production, and cellulase production [42,43,44,60,61,62]. Previous studies indicated that *C. neoformans CRK1* plays a negative role in cell fusion and pheromone expression in bisexual mating [29]. In this study, we provided a detailed observation for dikaryotic filamentation and demonstrated that the formation of basidia and basidiospores in the bilateral *crk1* mutant cross was earlier than the wild-type cross by approximately 18 h. The length of dikaryotic filaments in the bilateral *crk1* mutant cross was shorter but still maintained typical fused clamp cells and normal nuclear distribution. Taken together, our results revealed that *C. neoformans CRK1* was involved in maintaining proper dikaryotic filamentation and modulating the occurrence of karyogamy and meiosis to regulate accurate chronology in bisexual mating process. By bioinformatic search for the *C. neoformans* genes containing Ime2 consensus phosphorylation RPGT^1164^ site, we identified the *GAT1* gene as a putative target of *C. neoformans* Crk1. Our functional studies revealed that *GAT1* played a negative role in the transcription of *MAT2* and pheromone production, and the *crk1gat1* double mutant strain phenotypically copied the mating phenotypes of the *crk1* mutant in bisexual differentiation. Mutations of RPGT^1164^ in Gat1 resulted in dramatic phenotypes of bisexual mating, indicating that RPGT^1164^ sequence played an important function of Gat1 and was essential for proper regulation of bisexual mating. We demonstrated that both *C. neoformans CRK1* and *GAT1* formed a regulatory circuit to negatively modulate *MAT2* and other potential unidentified targets for pheromone production and other processes in bisexual mating.

It is interesting to observe shorter dikaryotic filaments and earlier basidium formation associated with the crosses involved *crk1* mutants. Early basidium formation was also reported in the *C. neoformans* mutant deleting the microtubule binding *BIM1* gene; however, mutation of *BIM1* also results in loss of filament integrity [63]. In contrast to the *bim1* mutant, the features of dikaryotic filaments in the bilateral *crk1* mutant cross, such as straight filament and typical clamp cell, did not differ from those in the wild-type cross (Figure 1). Moreover, the localization and distribution of nuclei in the bilateral *crk1* mutant cross were the same as the wild-type cross. These findings suggest that the involvement of *C. neoformans CRK1* gene in sexual development might be different from the *BIM1 gene*.

Mitotic progression is one of the mechanisms affecting cell size or filamentation in fungi [64,65]. Studies of the cell cycle machinery in *S. pombe* provide some insights for our study [66]. The *S. pombe wee1* gene is a mitotic inhibitor that controls G2/M transition. Deletion of the *wee1* gene decreases the period in G2 phase and causes early entry into mitosis, thus the cell size of *wee1* mutant is smaller than the wild-type [67]. Recently, the *S. pombe wee1* homologues were identified in other fungi, such as *U. maydis wee1* and *Beauveria bassiana wee1*. Mutation of *wee1* results in short filaments in *U. maydis* and *B. bassiana* [68,69]. Furthermore, *S. pombe cdc13* gene is an important B-type cyclin that promotes G2 phase to enter mitosis during cell cycle [70,71]. In *U. maydis*, overexpression of *clb2,* the *S. pombe cdc13* homologue, also results in shorter G2 phase and smaller cell size [72]. We assume that *C. neoformans* Crk1 may also regulate cell cycle transition. Preliminary bioinformatic survey in *C. neoformans* genome revealed the *wee1* homologues, CNG02130 and CNG03960, and several putative kinases which are related to cell cycle progression may be potential substrates of Crk1. The phenotypes exhibited by the *crk1* mutants may be caused by unscheduled cell-cycle progression. Whether Crk1 is involved in cell cycle progression in *C. neoformans* and how it connects to the sexual processes such as dikaryotic filamentation and basidium formation require further investigation.

The expression of *CRK1* is lower during early stage of bisexual mating process [29]. We confirmed similar results in this study and also found the *CRK1* mRNA level was slightly elevated between 16 to 18 h post-incubation (Figure 3). Thus, *CRK1* may play a significant role during later stage of mating and dikaryotic filamentation stage. To further reveal the role of *CRK1* in the bisexual mating process, we determined the expression of genes related to mating, filamentation, karyogamy, and meiosis in the bilateral *crk1* mutant cross. The expression of these genes was increased in the bilateral *crk1* mutant cross, which is consistent with previous conclusion that *CRK1* has a negative role in bisexual mating. Furthermore, as the *CRK1* mRNA level increased around 18 h in the wild-type cross, the expression of all the genes tested was also increased greatly at this stage in the bilateral *crk1* mutant cross (Figure 3). Elevated mRNA levels of *KAR7* and *DMC1* supported the phenotypic observation of early initiation of karyogamy and meiosis *in* the bilateral *crk1* mutant cross (Figure 1 and Figure 3E,F) These findings indicated that the *C. neoformans CRK1* gene might regulate multiple signal transduction events in bisexual mating.

*MAT2* and *ZNF2* are two major transcriptional regulators that control cell fusion and filamentation in *C. neoformans*. We further addressed the genetic relationship between *CRK1*, *MAT2* and *ZNF2*. It was hypothesized that the deletion of the negative regulator *CRK1* could bypass the non-filamentation phenotype in the bilateral *mat2* or *znf2* mutant cross. Interestingly, dikaryotic filamentation was blocked in the bilateral *znf2crk1* double mutant cross (Supplementary Figure S2A). However, the expression level of *CRK1* was not altered in the bilateral *znf2* mutant cross (Supplementary Figure S2C). These findings suggested that *C. neoformans ZNF2* possibly functioned downstream of *CRK1* in bilateral mating, thus resulting in non-filamentation phenotype in the bilateral *crk1znf2* mutant cross. In addition, dikaryotic filamentation was also blocked in the bilateral *mat2crk1* mutant cross; however, the expression of *CRK1* was decreased significantly in the bilateral *mat2* mutant cross and increased in the *MAT2*-overexpressed strain cross (Figure 4 and Figure 5D). In *S. cerevisiae*, *IME1* activates the expression of *IME2* at the initiation stage of meiosis, and then phosphorylation of Ime1 by Ime2 leads to the degradation of Ime1 for the completion of meiotic process [73,74,75]. Previous study showed that *C. neoformans* Mat2 regulates pheromone responsive genes by binding to the pheromone response element (PRE; AAAGAACAAAAAGACA) in their promoter [5]. An example is the *C. neoformans GPA3* gene which contains one PRE element at its promoter sequence and acts as a negative regulator of sexual differentiation [5]. We searched 1000 bp region upstream the *CRK1* open reading frame for the enriched motifs by the MEME algorithm, and one putative PRE (GGAAAAGAAAAGGTAT) was identified in the *CRK1* promoter. Along with the analysis of *CRK1* promoter sequence and our gene expression data, these findings suggest that *MAT2* might regulate *CRK1* expression during bisexual mating process.

*C. neoformans* Crk1 is a Ser/Thr protein kinase containing TXY activation motif. In this study, we predicted the potential targets of *C. neoformans* Crk1 based on Ime2 consensus phosphorylation site and the GATA transcription factor Gat1 was identified and characterized. The bilateral mutant crosses involved the *gat1* deletion mutants showed increased aerial hyphal formation and elevated expression of mating-related genes (Figure 6A and Figure 7). Strains containing the phospho-null *GAT1* allele in the predicted Ime2 consensus phosphorylation site also showed similar phenotypes as the *gat1* deletion mutants (Figure 6B). Our genetic and phenotypic findings indicated that *C. neoformans GAT1* gene modulated the sexual processes such as regulation of pheromone expression and aerial hyphae formation, which are overlapped with the roles of *CRK1* during bisexual mating. Interestingly, *GAT1* and *CRK1* played divergent role in basidia formation, as the deletion of *C. neoformans GAT1* gene did not result in early appearance of basidia during bisexual mating. In addition, *C. neoformans* wild-type and *crk1* mutant strains containing the *GAT1* phospho-mimetic allele both exhibited reduction of dikaryotic filamentation (Figure 9) and gene expression results also supported the roles of *GAT1* during bisexual mating (Figure 10). Thus, our data supported that *C. neoformans* Gat1, as one of the phosphorylation substrates of Crk1, also played a negative role in bisexual mating.

In our expression data, the transcript levels of the HMG transcription factor *MAT2* and *MF*α pheromone genes were both elevated in the bilateral *crk1* and *gat1* mutant crosses (Figure 3 and Figure 7). Moreover, *CRK1* was downregulated in the bilateral *mat2* mutant cross (Figure 4C). Studies in *S. pombe* indicated that the HMG family transcription factor *ste11* is a key regulator during sexual differentiation [56,76]. Under nitrogen starvation, the *S. pombe* GATA type transcription factor Gaf1, the homologue of *C. neoformans GAT1* gene, downregulates the expression of *ste11* via binding to the *ste11* promoter region. Deletion of *gaf1* results in increased *ste11* mRNA level [55]. Fungal GATA type transcription factors have also been demonstrated to recognize and bind to the consensus sequence, 5′-(T/A/C) GATA(A/G)-3′, of the promoter regions under regulation [76,77]. We initially analyzed and identified one putative binding site (5′-CGATAA-3′) in the 1 Kb upstream region of the *MAT2* promoter sequence. Thus, we hypothesize that *CRK1* was transcriptionally activated by *MAT2,* then Crk1 phosphorylated and activated Gat1, and then Gat1 repressed the expression of *MAT2* during bisexual mating process in *C. neoformans*. However, further studies for the evidences of the transcriptional and translational regulation are needed to support this regulatory circuit.

**Figure 11:**
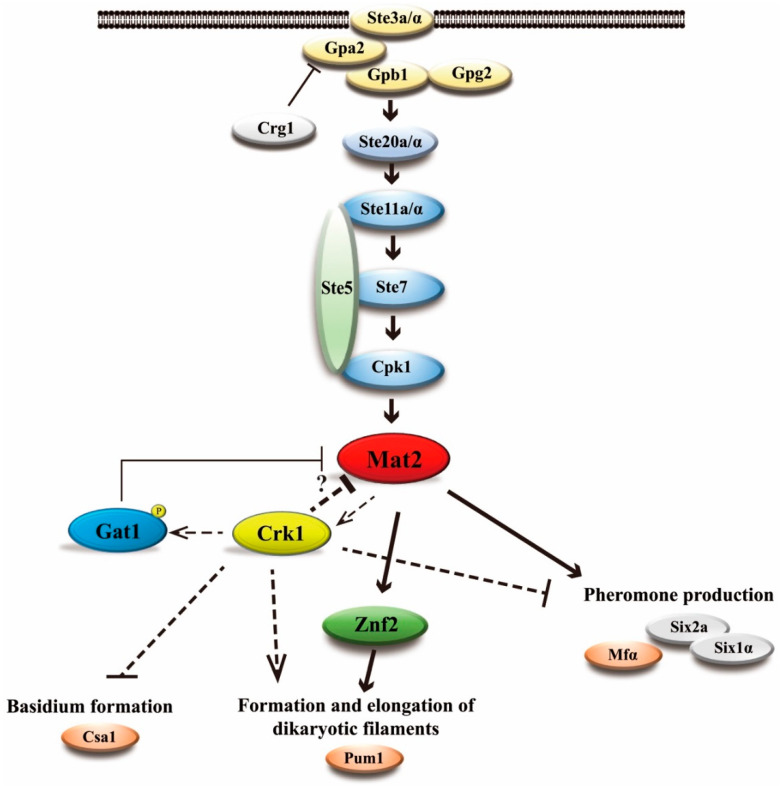
*C. neoformans* Crk1 and Gat1 negatively regulated the expression of Mat2 to coordinately modulate sexual differentiation. Mat2 is a major transcription factor downstream the Cpk1-MAPK signaling pathway to regulate pheromone production and mating responses in *C. neoformans*. The protein kinase Crk1 negatively regulated *MAT2* expression via phosphorylating Gat1 or other target(s) to reduce the *MAT2* transcript level and repress pheromone production and other mating responses. The expression of *CRK1* was possibly also regulated by Mat2. Crk1-Gat1 may form a regulatory circuit to properly regulate *MAT2*/Mat2 levels to control the progression of sexual filamentation and transition to basidium stage. We propose this model how Crk1 regulates pheromone production, elongation of dikaryotic filaments, and basidium formation during bisexual mating.

Based on our studies, we propose a potential regulatory circuit to illustrate how Crk1 and Gat1 regulate bisexual mating via the key transcriptional regulator Mat2 in *C. neoformans* (Figure 11). *C. neoformans CRK1* gene regulates pheromone production, formation and elongation of dikaryotic filaments and basidium formation. During the bisexual mating process, a pheromone is first sensed and transduced through the Cpk1-MAPK signaling pathway to activate the downstream transcription factor *MAT2. MAT2* plays a key role to modulate various sexual events including promoting cell fusion process and regulating *ZNF2* expression to induce dikaryotic filamentation. Based on our studies, we propose a regulatory circuit to describe how Crk1-Gat1 modulate *MAT2* activity for mating regulation. Upon pheromone induction, *MAT2* is activated via conserved signaling events to induce the expression of various genes including *CRK1*. Crk1 kinase regulates Gat1 activity by post-translational phosphorylation. The activated Gat1 will in turn inhibit *MAT2* expression to modulate bisexual mating processes (Figure 11). Since *GAT1* deletion and alleles with modified phosphorylation site could only partially suppress the filamentation phenotypes of *crk1* or wild-type cross (Figure 9), we hypothesize that *GAT1* possibly is not the only downstream target of Crk1 for mating regulation. Future study is needed to further elucidate the complicated regulatory networks mediated by Crk1.

## Figures and Tables

**Table 1:** *Cryptococcus neoformans* strains used in this study.

Strains	Description	Reference
JEC20	*MAT* **a**	[50]
JEC21	*MAT*α	[50]
JEC43	*MAT*α *ura5*	[51]
DLC2	*MAT*α *crk1*::*NAT*^R^	[29]
DLC4	*MAT***a***crk1*::*NAT*^R^	[29]
DLC18	*MAT***a***crk1* + P*_GPD1_*::*GFP-H2B ura5*	[29]
DLC21	*MAT***a** P*_GPD1_*::*GFP-H2B ura5*	[29]
DLC22	*MAT*α*crk1* + P*_GPD1_*::*CRK1*	This study
DLC23	*MAT***a***mat2*::*HYG*^R^	This study
DLC24	*MAT*α *mat2*::*HYG*^R^	This study
DLC25	*MAT***a***mat2*::*HYG*^R^*crk1*::*NAT*^R^	This study
DLC26	*MAT*α *mat2*::*HYG*^R^ *crk1*::*NAT*^R^	This study
DLC27	*MAT*α P*_GPD1_*::*MAT2*	This study
DLC28	*MAT***a***znf2*::*HYG*^R^	This study
DLC29	*MAT*α *znf2*::*HYG*^R^	This study
DLC30	*MAT***a***znf2*::*HYG*^R^*crk1*::*NAT*^R^	This study
DLC31	*MAT*α *znf2*::*HYG*^R^ *crk1*::*NAT*^R^	This study
DLC32	*MAT***a***gat1*::*HYG*^R^	This study
DLC33	*MAT*α *gat1*::*HYG*^R^	This study
DLC34	*MAT***a***gat1* + *GAT1*::*NAT*^R^	This study
DLC35	*MAT*α *gat1* + *GAT1*::*NAT*^R^	This study
DLC36	*MAT*α P*_GPD1_*::*GAT1*	This study
DLC37	*MAT*α *crk1* + P*_GPD1_*::*CRK1*	This study
DLC38	*MAT*α *crk1* + P*_GPD1_*::*CRK1* + P*_GPD1_*::*GAT1*	This study
DLC39	*MAT***a***GAT1*^T1164A^::*HYG*^R^	This study
DLC40	*MAT*α *GAT1*^T1164A^::*HYG*^R^	This study
DLC41	*MAT***a***crk1*::*NAT*^R^*gat1*::*HYG*^R^	This study
DLC42	*MAT*α *crk1*::*NAT*^R^ *gat1*::*HYG*^R^	This study
DLC43	*MAT***a** P*_GPD1_*::*GAT1*	This study
DLC44	*MAT***a***GAT1*^T1164D^::*HYG*^R^	This study
DLC45	*MAT*α *GAT1*^T1164D^::*HYG*^R^	This study
DLC46	*MAT***a***crk1* + *GAT1*^T1164D^::*HYG*^R^	This study
DLC47	*MAT*α *crk1* + *GAT1*^T1164D^::*HYG*^R^	This study
DLC48	*MAT***a** P*_GPD1_*::*CRK1 gat1*::*HYG*^R^	This study
DLC49	*MAT*α P*_GPD1_*::*CRK1 gat1*::*HYG*^R^	This study

## Supplementary Materials

The following are available online at https://www.mdpi.com/2073-4425/11/6/669/s1; Figure S1. Dikaryotic filamentation was examined in the wild-type and bilateral *crk1* mutant crosses. Co-incubation of (**A**) *MAT***a** wild-type P*_GPD1_*::*GFP-H2B* and *MAT*α wild-type strains and (**B**) *MAT***a** *crk1* P*_GPD1_*::*GFP-H2B* and *MAT*α *crk1* mutants was conducted on V8 agar plates incubated at 26° in the dark. Colony edges of mating mixtures were photographed from 16 to 72 h post-incubation at 100× magnification. Figure S2. *ZNF2* mutation did not affect *CRK1* expression, but blocked dikaryotic filamentation in the bilateral *crk1* mutant cross. (**A**) *C. neoformans MAT***a** and *MAT*α strains were crossed as indicated. Mating was conducted on V8 agar plates incubated at 26 °C in the dark. Photos were taken 24 h post-incubation at 100× magnification. Bilateral crosses of the *MAT***a** and *MAT*α wild-type and *znf2* mutants were conducted. Samples were collected at 0, 6, 18 and 24 h post-incubation and subjected to gene expression analysis. *MF*α (**B**) and *CRK1* (**C**) expression during bisexual mating was examined by real-time qRT-PCR analysis. Triplicate reactions for each sample were conducted. Error bar represents the standard deviation from the mean of three replicates. The results were normalized to *C. neoformans GPD1* expression. (** indicates *p* < 0.005). Figure S3. The expression level of mating-related genes were upregulated slightly in the bilateral *crk1gat1* mating cross. Bilateral crosses involved the *MAT***a** and *MAT*α wild-type, *gat1*, *crk1,* and *crk1gat1* mutants were conducted on V8 agar plates and incubated at 26 °C in the dark. Samples were collected at 0, 18, and 24 h post-incubation. The expression of *MF*α (**A**), *MAT2* (**B**), *CSA1* (**C**), and *DMC1* (**D**) was examined by real-time qRT-PCR analysis. Triplicate reactions for each sample were conducted. Error bar represents the standard deviation from the mean of three replicates. The results were normalized to *C. neoformans GPD1* expression. (* indicates *p* < 0.05). Figure S4. Overexpression of *GAT1* did not repress dikaryotic filamenation. *C. neoformans MAT***a** and *MAT*α strains *GAT1* overexpression strains were crossed as indicated. Mating was conducted on V8 agar plates at 26 °C in the dark. Photos were taken at 24 h post-incubation at 100× magnification. Figure S5. The transcription level of *MF*α in the *GAT1* overexpression strains cross was similar to that in the wild-type cross. *C. neoformans MAT***a** and *MAT*α wild-type and *GAT1* overexpression strains were crossed and incubated on V8 agar plates at 26 °C in the dark. Samples were collected at 0 and 24 h post-incubation. The expression of *GAT1* (**A**) and *MF*α (**B**) was examined by real-time qRT-PCR analysis. Triplicate reactions for each sample were conducted. Error bar represents the standard deviation from the mean of three replicates. The results were normalized to *C. neoformans GPD1* expression. Figure S6. Dikaryotic filamentation was reduced with overexpression of *GAT1* and *CRK1*. *C. neoformans MAT***a** and *MAT*α strains were crossed as indicated. Mating was conducted on V8 agar plates at 26 °C in the dark. Photos were taken at 24 h post-incubation at 100× magnification. Figure S7. Deletion of *GAT1* partially recovered sexual filamentation of bilateral *CRK1* overexpression cross. *C. neoformans MAT***a** and *MAT*α strains were crossed as indicated. Mating was conducted on V8 agar plates at 26 °C in the dark. Photos were taken at 24 h post-incubation at 100× magnification. Figure S8. *GAT1* negatively regulated *MF*α and *MAT2* gene expression during mating process. Bilateral crosses involved the *MAT***a** and *MAT*α wild-type, *gat1* mutants, P*_GPD1_*::*CRK1* overexpression strains, and P*_GPD1_*::*CRK1* + *gat1* mutant strains were conducted on V8 agar plates at 26 °C in the dark. Samples were collected at 0, 18, and 24 h post-incubation. The expression of *MF*α (**A**) and *MAT2* (**B**) was examined by real-time qRT-PCR analysis. Error bar represents the standard deviation from the mean of three replicates.Triplicate reactions for each sample were conducted. The results were normalized to *C. neoformans GPD1* expression. (* indicates *p* < 0.05; * indicates *p* < 0.005). Figure S9. Overexpression of *GAT1* and *CRK1* reduced *MF*α expression during bisexual mating. Bilateral crosses involved the *MAT***a** and *MAT*α wild-type, *crk1*, *gat1*, and *GAT1*^T1164A^ mutants, and *MAT***a** wild-type crossed with *MAT*α *crk1* + P*_GPD1_::CRK1*, and *MAT*α *crk1 +* P*_GPD1_::CRK1 +* P*_GPD1_::GAT1* strains were conducted on V8 agar plates at 26 °C in the dark. Samples were collected at 0 and 24 h post-incubation and the expression of *GAT1* (**A**) and *MF*α (**B**) was examined by real-time qRT-PCR analysis. Triplicate reactions for each sample were conducted. Error bar represents the standard deviation from the mean of three replicates. The results were normalized to *C. neoformans GPD1* expression. Table S1. Oligonucleotides used in this study. Table S2. Potential transcription factor targets of *C. neoformans* Crk1.
