# Supplementary material for: Sexual Differentiation Is Coordinately Regulated by Cryptococcus neoformans CRK1 and GAT1
Source: Genes (Basel). 2020 Jun 19;11(6):669. doi: 10.3390/genes11060669 (PMC7349709; doi:10.3390/genes11060669)
Supplement: Supplementary file 1 [file genes-11-00669-s001.pdf]

Supplementary Materials: Sexual Differentiation Is Coordinately Regulated by *Cryptococcus neoformans* *CRK1* and *GAT1*

Kuang-Hung Liu and Wei-Chiang Shen

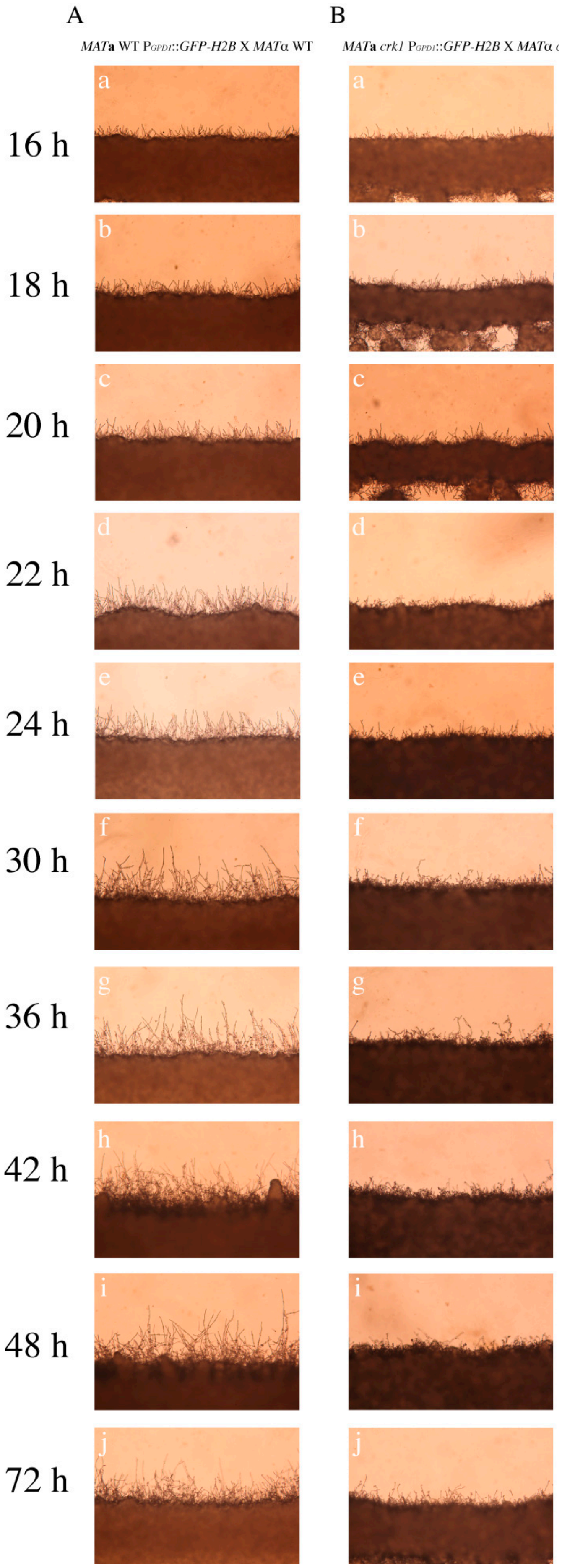
A
B
MATa WT PGPD1::GFP-H2B X MATα WT
MATa crk1 PGPD1::GFP-H2B X MATα
16 h
18 h
20 h
22 h
24 h
30 h
36 h
42 h
48 h
72 h
a
b
c
d
e
f
g
h
i
j

Figure S1. Dikaryotic filamentation of the wild-type and bilateral *crk1* mutant crosses during mating process was examined. Bisexual mating of the *MAT***a** wild-type P_{GPD1}::*GFP-H2B* and *MAT*α wild-type strains (**A**) and *MAT***a** *crk1* P_{GPD1}::*GFP-H2B* and *MAT*α *crk1* mutants (**B**) was conducted on V8 agar plates incubated at 26° in the dark. Colony edges of mating mixtures were photographed from 16 to 72 hr post-incubation at 100x magnification.

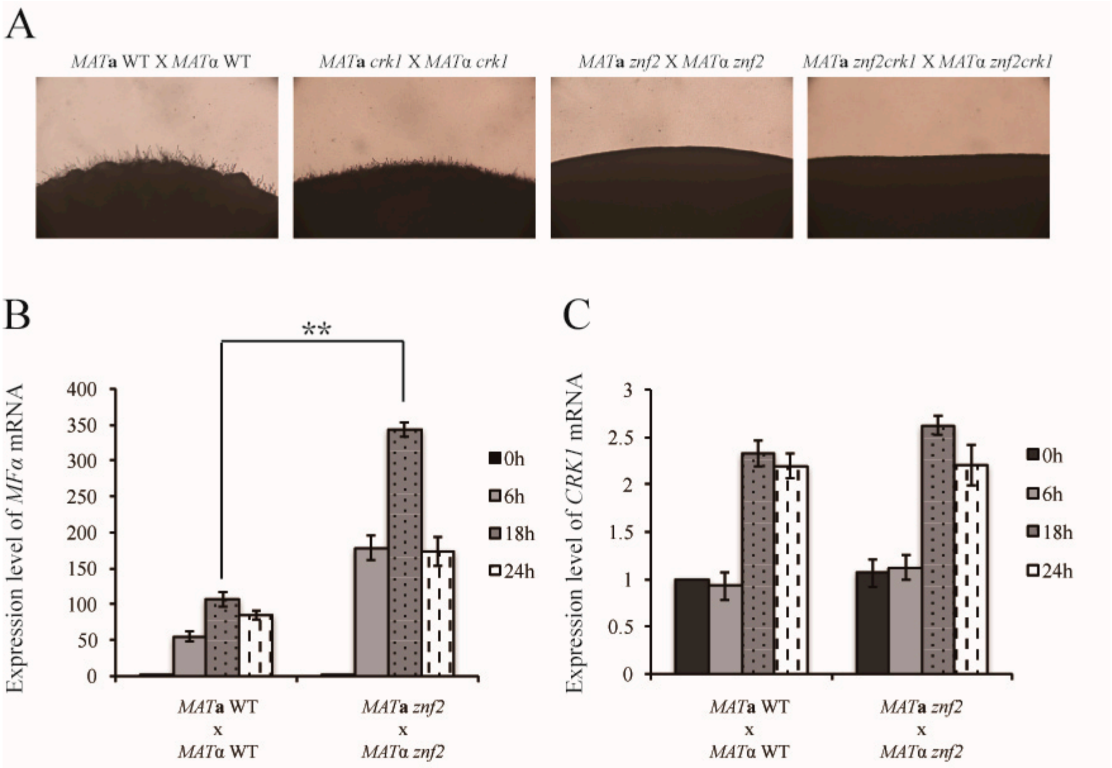

Figure S2. *ZNF2* mutation did not affect *CRK1* expression, but blocked dikaryotic filamentation in the bilateral *crk1* mutant cross. (**A**) *C. neoformans MAT***a** and *MAT*α strains were crossed as indicated. Mating was conducted on V8 agar plates incubated at 26° in the dark. Photos were taken 24 hr post-incubation at 100x magnification. Bilateral crosses of the *MAT***a** and *MAT*α wild-type and *znf2* mutants were conducted. Samples were collected at 0, 6, 18 and 24 hr post-incubation and subjected to gene expression analysis. *MF*α (**B**) and *CRK1* (**C**) expression during bisexual mating was examined by real-time qRT-PCR analysis. Triplicate reactions for each sample were conducted. The results were normalized to *C. neoformans GPD1* expression. (** indicates P<0.005).

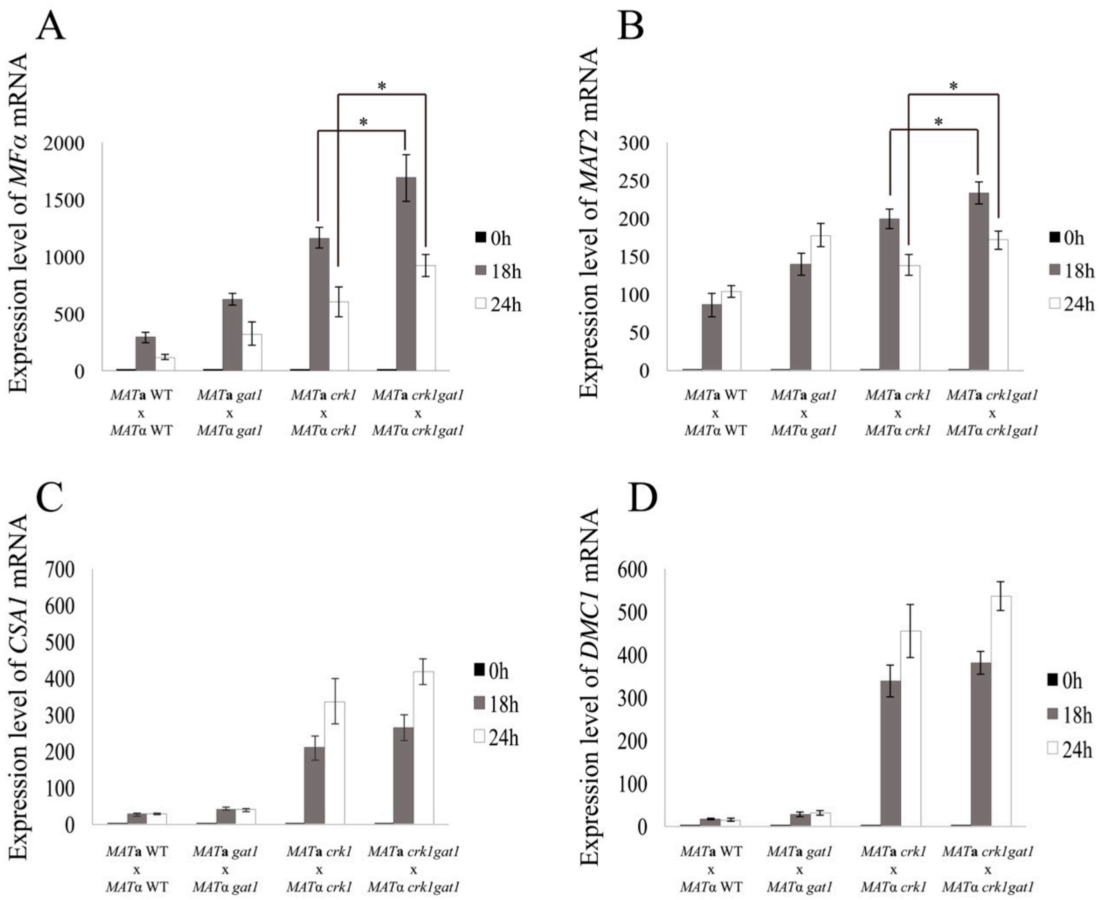

Figure S3. The expression level of mating-related genes were upregulated slightly in the bilateral *crk1gat1* mating cross. Bilateral crosses involved the *MAT***a** and *MAT*α wild-type, *gat1*, *crk1,* and *crk1gat1* mutants were conducted on V8 agar plates and incubated at 26° in the dark. Samples were collected at 0, 18, and 24 hr post-incubation. The expression of *MF*α (**A**), *MAT2* (**B**), *CSA1* (**C**), and *DMC1* (**D**) was examined by real-time qRT-PCR analysis. Triplicate reactions for each sample were conducted. The results were normalized to *C. neoformans GPD1* expression. (* indicates P< 0.05).

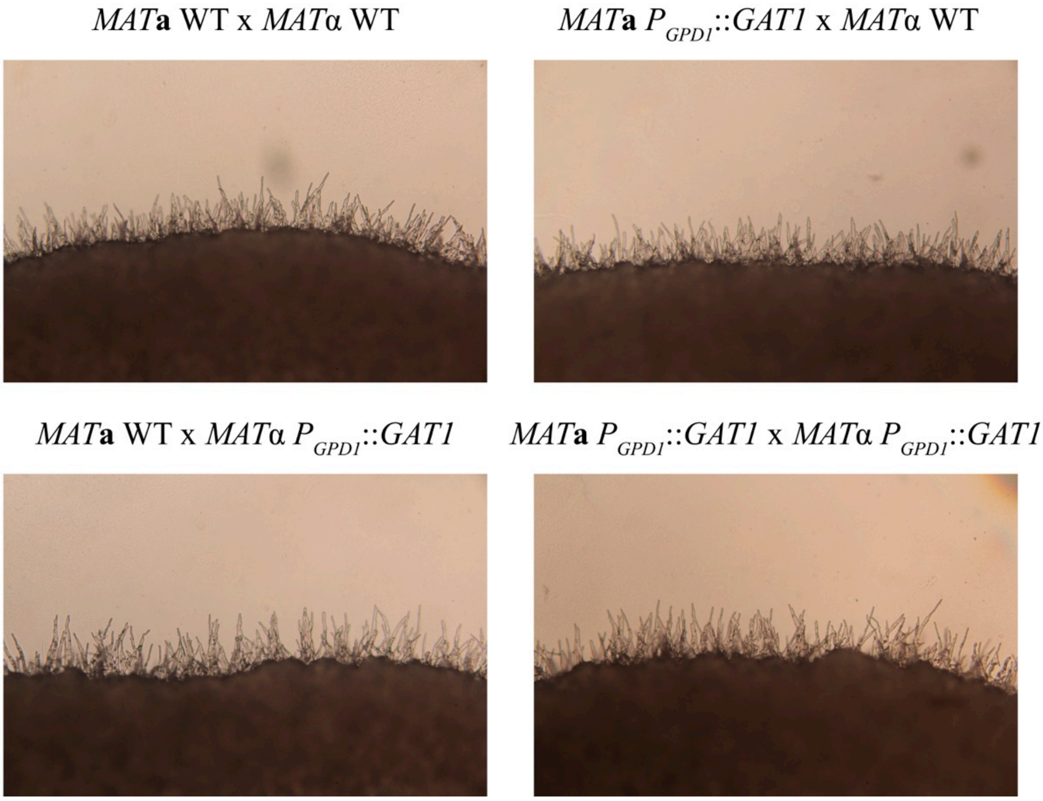

Figure S4. Overexpression of *GAT1* did not repress dikaryotic filamenation. *C. neoformans MAT***a** and *MAT*α strains *GAT1* overexpression strains were crossed as indicated. Mating was conducted on V8 agar plates at 26° in the dark. Photos were taken at 24 hr post-incubation at 100x magnification.

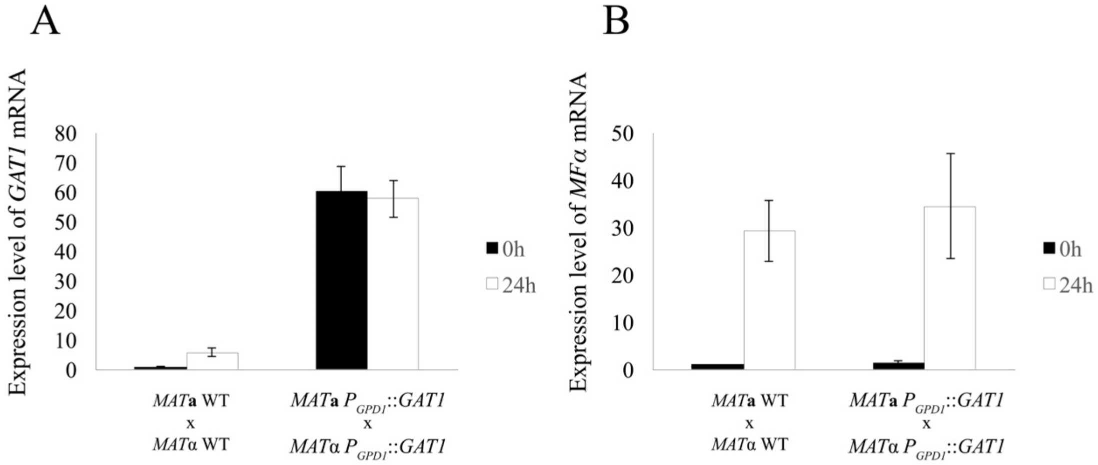

Figure S5. The transcription level of *MF*α in the *GAT1* overexpression strains cross was similar to that in the wild-type cross. *C. neoformans MAT***a** and *MAT*α wild-type and *GAT1* overexpression strains were crossed and incubated on V8 agar plates at 26° in the dark. Samples were collected at 0 and 24 hr post-incubation. The expression of *GAT1* (**A**) and *MF*α (**B**) was examined by real-time qRT-PCR analysis. Triplicate reactions for each sample were conducted. The results were normalized to *C. neoformans GPD1* expression.

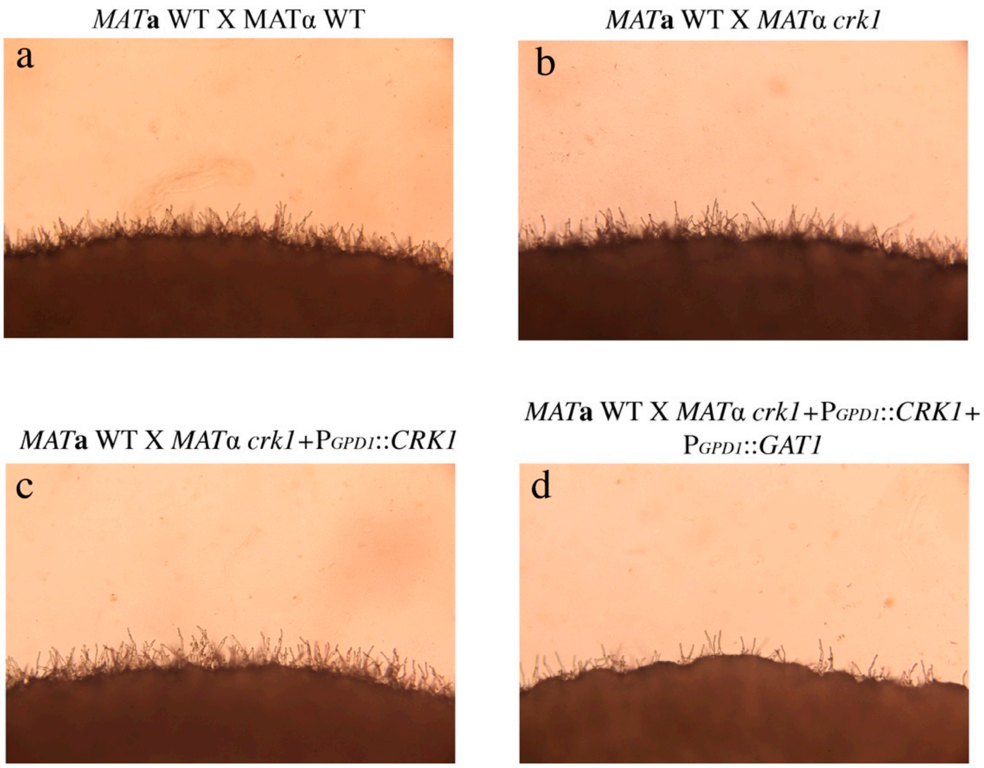

Figure S6. Dikaryotic filamentation was reduced with overexpression of *GAT1* and *CRK1*. *C. neoformans MAT***a** and *MAT*α strains were crossed as indicated. Mating was conducted on V8 agar plates at 26° in the dark. Photos were taken at 24 hr post-incubation at 100x magnification.

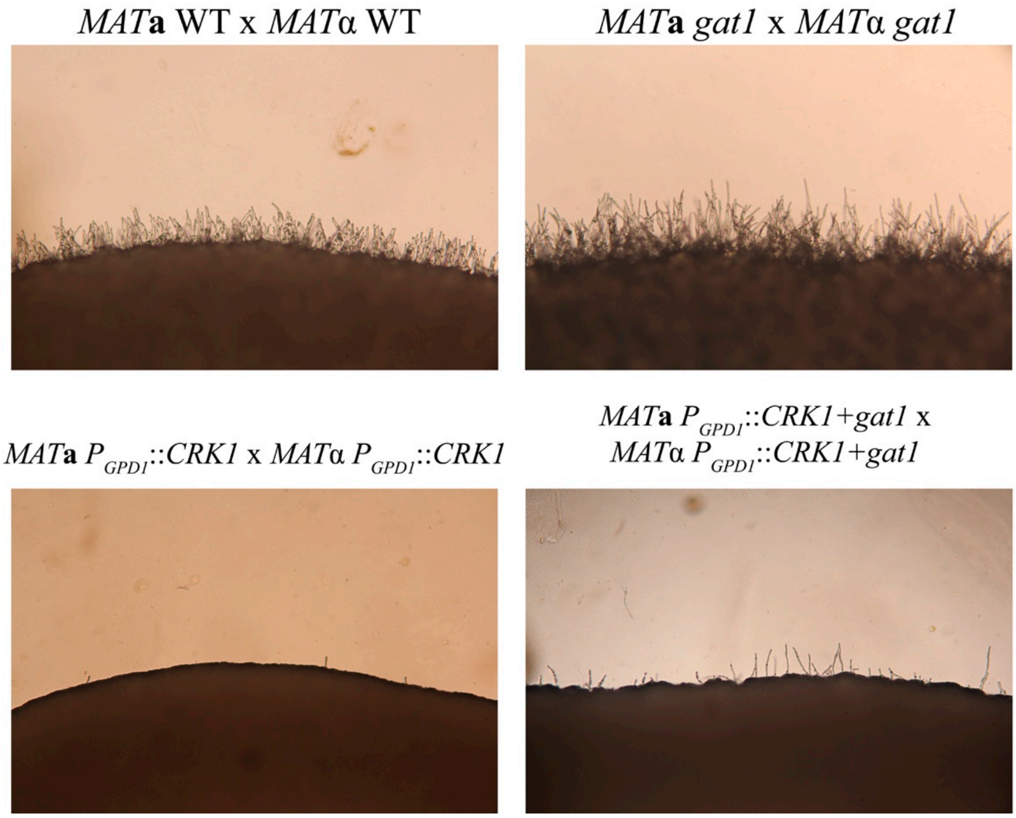

Figure S7. Deletion of *GAT1* partially recovered sexual filamentation of bilateral *CRK1* overexpression cross. *C. neoformans MAT***a** and *MAT*α strains were crossed as indicated. Mating was conducted on V8 agar plates at 26° in the dark. Photos were taken at 24 hr post-incubation at 100x magnification.

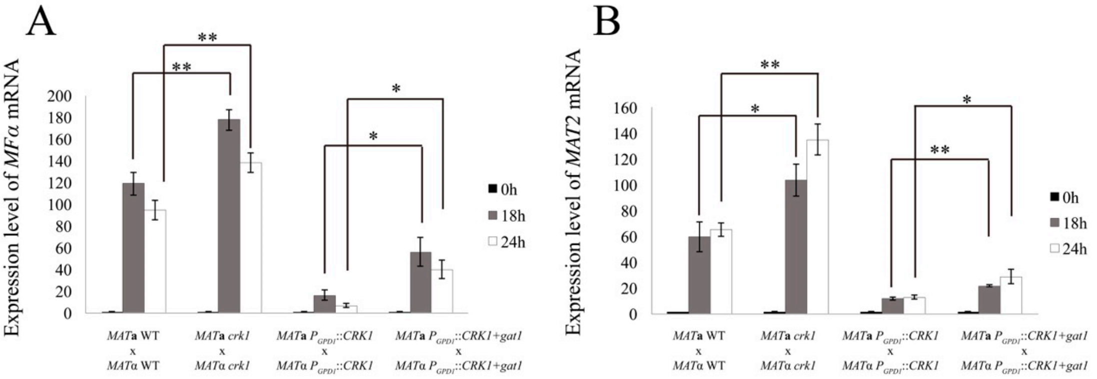

Figure S8. *GAT1* negatively regulated *MF*α and *MAT2* gene expression during mating process. Bilateral crosses involved the *MAT***a** and *MAT*α wild-type, *gat1* mutants, P$_{GPD1}$::*CRK1* overexpression strains, and P$_{GPD1}$::*CRK1+gat1* mutant strains were conducted on V8 agar plates at 26° in the dark. Samples were collected at 0, 18, and 24 hr post-incubation. The expression of *MF*α (A) and *MAT2* (B) was examined by real-time qRT-PCR analysis. Triplicate reactions for each sample were conducted. The results were normalized to *C. neoformans GPD1* expression. (* indicates P< 0.05; * indicates P< 0.005).

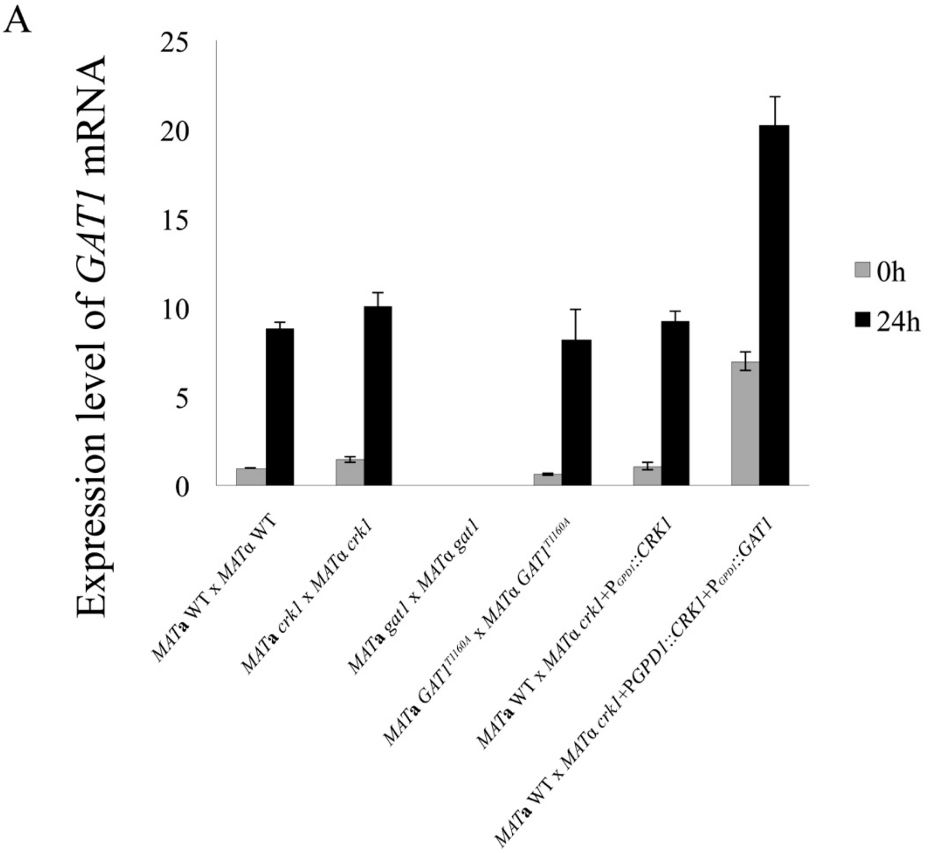

B

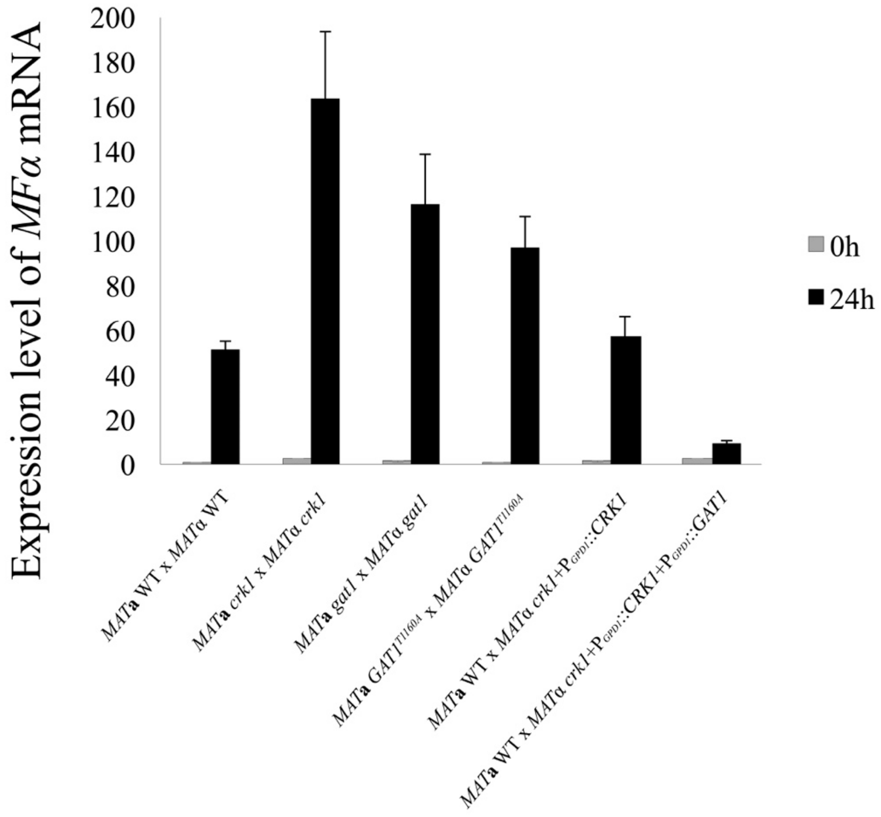

Figure S9. Overexpression of *GAT1* and *CRK1* reduced *MF*α expression during bisexual mating. Bilateral crosses involved the *MAT***a** and *MAT*α wild-type, *crk1*, *gat1*, and *GAT1*^{T1164A} mutants, and *MAT***a** wild-type crossed with *MAT*α *crk1* + P$_{GPD1}$::*CRK1*, and *MAT*α *crk1* + P$_{GPD1}$::*CRK1*+ P$_{GPD1}$::*GAT1* strains were conducted on V8 agar plates at 26° in the dark. Samples were collected at 0 and 24 hr post-incubation and the expression of *GAT1* (**A**) and *MF*α (**B**) was examined by real-time qRT-PCR analysis. Triplicate reactions for each sample were conducted. The results were normalized to *C. neoformans GPD1* expression.

Table S1. Oligonucleotide primers used in this study.

Primer name	Sequences (5'-3')	Description
WC270	GCTGCGAGGATGTGAGCTGG	HYG marker
WC271	GGTTTATCTGTATTAACACGG	HYG marker
WC739	GTATTGACCGATTCCTTGCGGTCCGAA	HYG marker
WC765	GATGTAGGAGGGCGTGGATATGTCCT	HYG marker
WC885	AAATAGCTGCGCCGATGGTTT	HYG marker
WC886	CGAACCCGCTCGTCTGGCTAA	HYG marker
WC1130	GCAGAGAACAGTTAGAAGCC	*MAT2* disruption
WC1131	CTTCCGTGTTAATACAGATAAACCATCTGATCGATACAA	*MAT2* disruption
WC1132	CTCTCCAGCTCACATCCTCGCAGCTGAGTGGTATACCCTA	*MAT2* disruption
WC1133	GTTGCGTGTCGAACCATCTT	*MAT2* disruption
WC1734	CGGGATCCATGCTCAAGAGAATTAGTGA	*MAT2* overexpression
WC1735	CGGGGTACCTGTTGGCTGTCACTGC	*MAT2* overexpression
WC1256	TTCTGAGCCATATATCTGTC	*ZNF2* disruption
WC1257	CTTCCGTGTTAATACAGATAAACCTGTCGTAAAGGATGGAGGAG	*ZNF2* disruption
WC1258	CTCTCCAGCTCACATCCTCGCAGCCGTTAATCCAAAGTATTGAC	*ZNF2* disruption
WC1259	CAAGACCTATAAAGCGAGAT	*ZNF2* disruption
WC2432	GTTGACCCTTCCGGGGTGT	*GAT1* disruption
WC2433	TTGCTACCGACAGTTTCCCT	*GAT1* disruption
WC2434	CTTCCGTGTTAATACAGATAAACCTCTAGCCGCCGCTAGCTGCT	*GAT1* disruption
WC2435	CTCTCCAGCTCACATCCTCGCAGCGCGAATGAATAGGAGCAGTG	*GAT1* disruption
WC2436	TCAACTGCTAACAGTCCGGA	*GAT1* disruption
WC2437	GCGCGGCGGCAGAAACTTTC	*GAT1* disruption
WC2555	GCGGCCGCATGGACAAGCTTCCATGGCGCAC	*GAT1* overexpression
WC2556	GCGGCCGCGTTGAAAAGAAAGGCGCACG	*GAT1* overexpression
WC2594	ACTAGTCCTCTCAAGCCCTACGTATT	*GAT1* reconstitution
WC2595	ACTAGTGTTGAAAAGAAAGGCGCACG	*GAT1* reconstitution
WC1181	TGTAAAACGACGGCCAG	*GAT1* site-directed mutagenesis
WC2567	GGATCCCAGCAAGCCGATCCCGTTCT	*GAT1* site-directed mutagenesis
WC2573	ACTAGTGTTGAAAAGAAAGGCGCACG	*GAT1* site-directed mutagenesis
WC2604	CCCGACCAGGTGCGCCGACGAGTGAA	*GAT1* site-directed mutagenesis
WC2605	TTCACTCGTCGGCGCACCTGGTCGGG	*GAT1* site-directed mutagenesis

WC2639	GTGAGTTACCGTGTCAACAT	*GAT1* site-directed mutagenesis
WC3257	CCCGACCAGGTGACCCGACGAGTGAA	*GAT1* site-directed mutagenesis
WC3258	TTCACTCGGGTCACCTGGTCGGG	*GAT1* site-directed mutagenesis
WC316	TTCTGAGAGCCCTGAGT	*GPD1* qRT-PCR
WC317	GGCATCAACACCAGCA	*GPD1* qRT-PCR
WC566	TGAACAGGGTGGAGAAAGGTAAG	*SXI1α* qRT-PCR
WC567	AGTGAAACGGTATTTGAAGGCG	*SXI1α* qRT-PCR
WC572	CTCGCTCATTAGACAGCAACTCA	*MFα* qRT-PCR
WC573	GAAGATGGCAGTGAAGGCGT	*MFα* qRT-PCR
WC617	AGTTGATGCTATGTTAGGTGGAGGA	*DMC1* qRT-PCR
WC618	CGCACAAGGTATGACAAAGCTG	*DMC1* qRT-PCR
WC875	CCAGATATCAGAGCGGTGTACG	*MAT2* qRT-PCR
WC876	TTTTCGGCCTTCCTCTTAGGT	*MAT2* qRT-PCR
WC879	GATGCTGCCGCTTGAAATG	*ZNF2* qRT-PCR
WC880	TCGCGAGACATAGGCGTATTC	*ZNF2* qRT-PCR
WC1428	CAGCAATGGCCATCTTTTCTC	*KAR7* qRT-PCR
WC1429	CGGTTCGTCAGCCAACAGA	*KAR7* qRT-PCR
WC1440	TATGCCTCCACCACCAGATGT	*CRK1* qRT-PCR
WC1441	GGCTGTCGGGTCTACCAATC	*CRK1* qRT-PCR
WC2133	AGGTGCAGAGGTTACGGTGATT	*GAT1* qRT-PCR
WC2134	TTGTCGAGCCTGGAGAATGC	*GAT1* qRT-PCR
WC2717	AGCCCGAGGACAGGAACAA	*PUM1* qRT-PCR
WC2718	CGTGAATGAGGGCCTTTTCA	*PUM1* qRT-PCR
WC3370	AGACTCGACCACAGGCAG	*CSA1* qRT-PCR
WC3371	AAAGGACAGGGTCAGGGTT	*CSA1* qRT-PCR

Table S2. Potential transcription factor targets of *C. neoformans* Crk1.

JEC21 ID	H99 ID	Gene Name
CNA01820	CNAG_00193	*GAT1*
CNA06480	CNAG_00670	*FZC12*
CNA07670	CNAG_00791	*HLH1*
CNB02070	CNAG_03710	*ECM22*
CNC02140	CNAG_01708	*GAT7*
CND02990	CNAG_01173	*PAN1*
CNG03610	CNAG_03212	*HCM101*
CNG04450	CNAG_03116	*HCM1*
CNH00870	CNAG_05420	*RGM1*
CNI01910	CNAG_04359	Hypothetical protein
CNI02050	CNAG_04345	*ARO8001*
CNJ00250	CNAG_04583	*DDT1*
CNJ00280	CNAG_04586	*HOB7*
CNJ00300	CNAG_04588	*ERT1*
CNJ00330	CNAG_04594	*FZC27*
CNJ00670	CNAG_04630	*YAP2*
CNJ02030	CNAG_04774	*FZC26*
CNJ02610	CNAG_04836	*FZC10*
CNJ03310	CNAG_04908	*CLR4*
CNK00590	CNAG_02566	*FKH2*
CNM01040	CNAG_06097	Hypothetical protein
CNM02250	CNAG_06223	*MIZ1*

© 2020 by the authors. Submitted for possible open access publication under the terms and conditions of the Creative Commons Attribution (CC BY) license (http://creativecommons.org/licenses/by/4.0/).
